# iCLIP analysis of RNA substrates of the archaeal exosome

**DOI:** 10.1186/s12864-020-07200-x

**Published:** 2020-11-16

**Authors:** Jochen Bathke, A. Susann Gauernack, Oliver Rupp, Lennart Weber, Christian Preusser, Marcus Lechner, Oliver Rossbach, Alexander Goesmann, Elena Evguenieva-Hackenberg, Gabriele Klug

**Affiliations:** 1 Institute of Microbiology and Molecular Biology, Justus-Liebig-University, 35392 Giessen, Germany; 2 Institute of Bioinformatics and Systems Biology, Justus-Liebig-University, 35392 Giessen, Germany; 3 Institute of Biochemistry, Justus-Liebig-University, 35392 Giessen, Germany; 4 Center for Synthetic Microbiology & Department of Pharmaceutical Chemistry, Philipps-University Marburg, 35032 Marburg, Germany

**Keywords:** Archaea, circRNA, Exosome, Exoribonuclease, iCLIP, Poly(A), RNA binding, *Sulfolobus solfataricus*

## Abstract

**Background:**

The archaeal exosome is an exoribonucleolytic multiprotein complex, which degrades single-stranded RNA in 3′ to 5′ direction phosphorolytically. In a reverse reaction, it can add A-rich tails to the 3′-end of RNA. The catalytic center of the exosome is in the aRrp41 subunit of its hexameric core. Its RNA-binding subunits aRrp4 and aDnaG confer poly(A) preference to the complex. The archaeal exosome was intensely characterized in vitro, but still little is known about its interaction with natural substrates in the cell, particularly because analysis of the transcriptome-wide interaction of an exoribonuclease with RNA is challenging.

**Results:**

To determine binding sites of the exosome to RNA on a global scale, we performed individual-nucleotide resolution UV crosslinking and immunoprecipitation (iCLIP) analysis with antibodies directed against aRrp4 and aRrp41 of the chrenarchaeon *Sulfolobus solfataricus*. A relatively high proportion (17–19%) of the obtained cDNA reads could not be mapped to the genome. Instead, they corresponded to adenine-rich RNA tails, which are post-transcriptionally synthesized by the exosome, and to circular RNAs (circRNAs). We identified novel circRNAs corresponding to 5′ parts of two homologous, transposase-related mRNAs. To detect preferred substrates of the exosome, the iCLIP reads were compared to the transcript abundance using RNA-Seq data. Among the strongly enriched exosome substrates were RNAs antisense to tRNAs, overlapping 3′-UTRs and RNAs containing poly(A) stretches. The majority of the read counts and crosslink sites mapped in mRNAs. Furthermore, unexpected crosslink sites clustering at 5′-ends of RNAs was detected.

**Conclusions:**

In this study, RNA targets of an exoribonuclease were analyzed by iCLIP. The data documents the role of the archaeal exosome as an exoribonuclease and RNA-tailing enzyme interacting with all RNA classes, and underlines its role in mRNA turnover, which is important for adaptation of prokaryotic cells to changing environmental conditions. The clustering of crosslink sites near 5′-ends of genes suggests simultaneous binding of both RNA ends by the *S. solfataricus* exosome. This may serve to prevent translation of mRNAs dedicated to degradation in 3′-5′ direction.

**Supplementary Information:**

The online version contains supplementary material available at 10.1186/s12864-020-07200-x.

## Background

*Sulfolobus solfataricus* is a crenarchaeon with a growth optimum at 80 °C to 85 °C and pH 2 to 4 [1, 2] and is a widely used model organism for analysis of RNA processing and degradation in the third domain of life [3]. As most archaea, it harbors a multiprotein complex for exoribonucleolytic degradation named the exosome [4, 5]. This complex is homologous to the trimeric bacterial polynucleotide phosphorylase (PNPase) and the eukaryotic exosome, both having essential functions in RNA metabolism. In the past, intense in vitro analyses provided valuable information on the structure, substrate binding and catalytic mechanism of the archaeal exosome [5, 6]. Despite this, still little is known about the natural substrates of the exosome.

The archaeal exosome has a conserved nine-subunit core comprised of homologs of the eukaryotic proteins Rrp41, Rrp42, Rrp4 and Csl4 ([4, 6]. The archaeal proteins aRrp41 and aRrp42 contain RNase PH domains and form a hexameric ring with three phosphorolytically active sites in aRrp41, which are located near the bottom of the hexamer [7]. The proteins aRrp4 and aCsl4 contain S1 and KH/Zn-finger domains and build a heterotrimeric RNA-binding cap on the top of the hexamer [6]. Structurally, this nine-subunit complex resembles bacterial PNPase and the nine-subunit core of the eukaryotic exosome [8, 9]. According to studies of reconstituted *S. solfataricus* exosomes, the two proteins of the trimeric cap have specific functions in interactions with other proteins and with RNA substrates: aRrp4 shows poly(A)-preference, while aCsl4 is needed for the tight binding of the archaea-specific exosomal subunit aDnaG [10, 11]. The protein aDnaG harbors a novel RNA-binding domain with poly(A) preference, thus enlarging the RNA-binding platform of the exosome [12]. Further, aDnaG (and the exosome) interacts with the Sm-like proteins SmAP1 and SmAP2, and this interaction seems to influence the subcellular localization of the exosome and the levels of the A-rich RNA tails in the cell [13]. Finally, the exosome was found to interact with aNop5 in the stationary growth phase, and this interaction depends on aRrp4 [14]. Nop5 is part of an RNA methylating protein complex [15].

Upon binding of a transcript with a single-stranded 3′-end to the RNA-binding cap of the archaeal exosome, the 3′-end is threaded through a narrow side (neck) in the central channel of the hexameric ring until it reaches the chamber with the three phosphorolytic active sites [7, 16–18]. Tight binding of the substrate at the neck is a prerequisite for fast RNA degradation, because in the phosphorolytic chamber the 3′-end of the RNA is released after each catalytic step and switches between the three active sites. RNA of 10 nt spans between an active site and the neck [19], and therefore smaller RNA fragments are degraded more slowly and in a distributive manner [20]. Interaction of RNA with the cap proteins may also contribute to the tight binding of the substrate to the phosphorolytic chamber, because when a labeled 30 nt poly(A) substrate is shortened to 25 nt, it can be outcompeted by longer, non-labeled substrates [10].

The phosphorolytic mode of action of the exosome explains its dual function as an exoribonuclease and polynucleotidyl-transferase. In a reaction reverse to phosphorolysis, the archaeal exosome uses rNDPs to add adenine-rich (A-rich) tails to the 3′-ends of RNAs, a function that was also described for PNPase [21, 22]. In contrast, the nine-subunit core of the exosome in yeast and human is catalytically inactive, and the (hydrolytic) ribonuclease activity is exerted by additional subunit(s) (Rrp44/Dis3 and Rrp6) [23]. The RNA tails added posttranscriptionally increase degradation of structured RNA in vitro by the archaeal exosome and most probably, they also destabilize RNA in vivo [24]. The exosome is a major exoribonuclease and the only RNA-tailing enzyme in archaea [21], and is expected to participate in maturation and degradation of essentially all RNAs in the cell. However, its substrates are not studied at the global level yet.

To date, transcriptome-wide analyses of RNase substrates were performed mostly for bacterial endoribonucleases as RNA-Seq comparisons between wild type and mutant strains [25–28] Recently, this approach was also applied to study the targetomes of 3′-5′ exoribonucleases in *Streptococcus pyogenes* [29]. Furthermore, crosslinking and immunoprecipitation (CLIP), followed by RNA sequencing (RNA-Seq), was used to study the interactome of RNA binding proteins and to identify sRNA-mRNA interactions in bacteria [30, 31]. Archaeal RNases were not analyzed by CLIP yet.

In this study, the iCLIP (individual-nucleotide resolution UV crosslinking and immunoprecipitation) method [32–34] was used to detect RNAs bound to the archaeal exosome in *S. solfataricus*. Our data show that the archaeal exosome interacts with mRNAs, housekeeping non-coding RNAs, antisense and circular RNAs, and with posttranscriptionally added RNA tails. Importantly, our results suggest that during the exoribonucleolytic degradation in 3′-5′ direction, the exosome interacts with the 5′-end of its RNA substrates.

## Results

### iCLIP of *S. solfataricus* with antibodies directed against aRrp41 and aRrp4

**Fig. 1:**
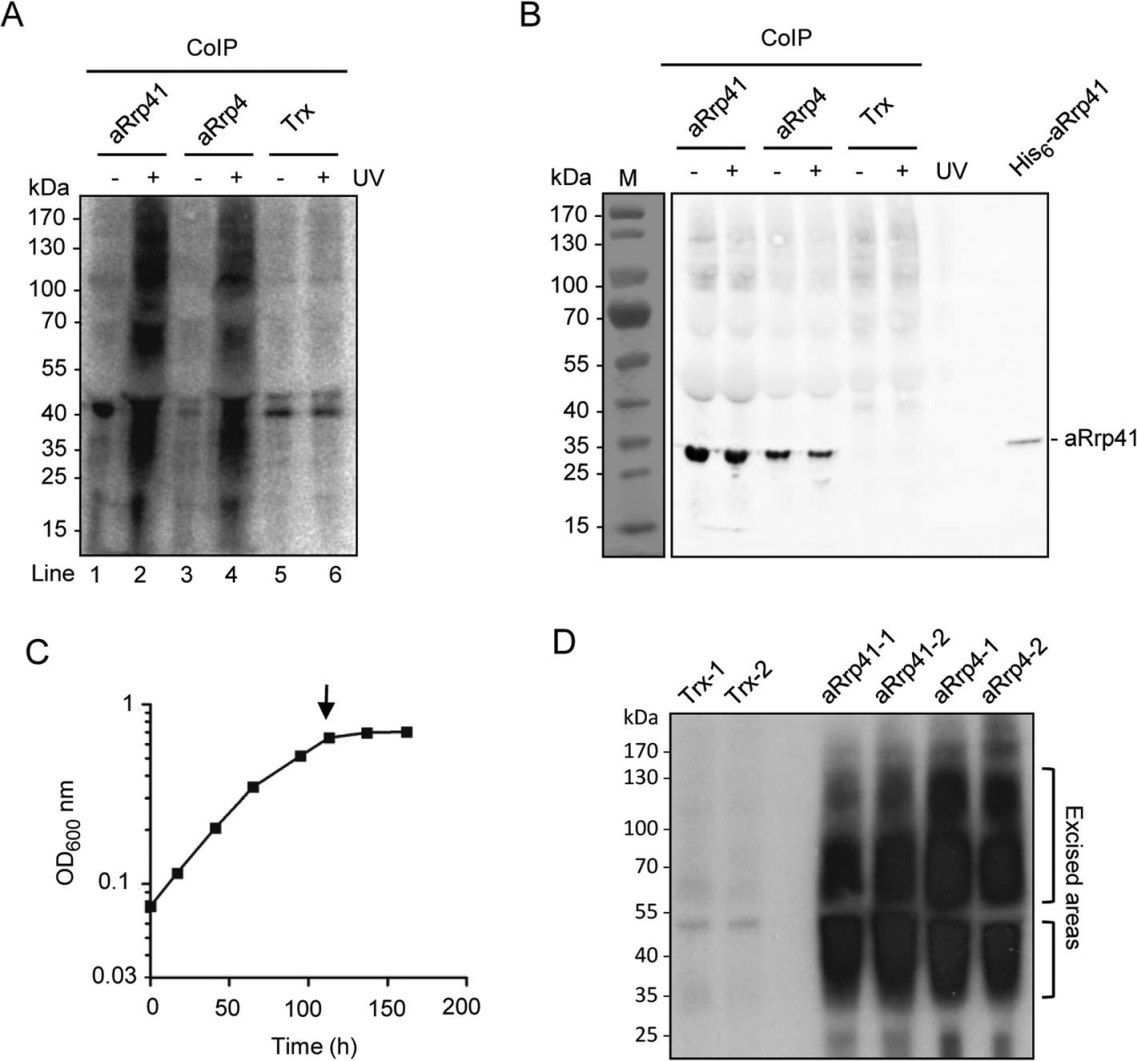
Isolation of RNA crosslinked to the exosome of *S. solfataricus*. **a** Autoradiogram of a nitrocellulose membrane with transferred, coimmunoprecipitated proteins, attached to crosslinked, radioactively labeled RNA. Archaeal cells were subjected to UV irradiation (+ UV) or not (− UV) to crosslink RNA to RNA binding proteins in vivo. After lysis, the cleared lysate was used for CoIP with antibodies directed against the proteins indicated above. After extensive washing with buffer containing 1 M NaCl, the coimmunoprecipitated, crosslinked RNA was radioactively labeled directly on the beads. The coimmunoprecipitated proteins with crosslinked RNA were separated by SDS-PAGE and transferred to the membrane. The uncropped image is shown in Fig. S1 (Additional file 1). **b** Western blot analysis of the nitrocellulose membrane shown in **a**) with antibodies directed against aRrp41. On the right side of the gel, recombinant, His_6_-tagged aRrp41 was loaded as positive control (cropped in panel **a**; see Fig. S1). Here, a non-blotted gel lane with marker proteins (run in the same gel) is shown on the left. **c** Representative growth curve of *S. solfataricus* in a 10 l bioreactor. Cells were harvested for iCLIP at OD_600_ of 0.7 (marked with an arrow). **d** Autoradiogram of the nitrocellulose membrane with samples used in our iCLIP analysis. Harvested cells were UV-irradiated, divided into 3 portions and subjected to CoIP with antibodies specific to aRrp41, aRrp4 and Trx. Two biological replicates were performed. After CoIP, 3′-RNA linker ligation and radioactive labeling of bound RNA, protein-RNA complexes were separated by SDS-PAGE and transferred to a nitrocellulose membrane. The autoradiogram was used to determine the membrane areas of the protein/RNA complexes (marked on the right side), which were excised and used for iCLIP library preparation and sequencing. The uncropped image is shown in Fig. S1 (Additional file 1)

An iCLIP experiment was performed with antibodies directed against aRrp41 and aRrp4 of *S. solfataricus*. Previous studies revealed very tight interaction between the exosomal subunits, which withstands washing with 1 M NaCl during the co-immunoprecipitation (CoIP) procedure [35]. Thus, we decided to include 1 M NaCl in the washing buffer of the iCLIP experiment to avoid non-specific interactions. To test the UV crosslinking of RNA to the exosome in *S. solfataricus* cells, harvested cells were resuspended and divided into two halves: one half was irradiated with UV, and the second was not. After lysis by sonification, each of the two cleared lysates was divided in three portions for CoIP with three different polyclonal antibodies. In addition to the aRrp41- and aRrp4-directed antibodies [36], antibodies against thioredoxin (Trx) from the alphaproteobacterium *R. sphaeroides* [37] were used as a negative control, since this protein is not expected to bind RNA. After the binding and washing procedure, co-precipitated RNA was labeled with ^32^P, and proteins with crosslinked, radioactively labeled RNAs were detected by autoradiography following SDS-PAGE and transfer to a nitrocellulose membrane (Fig. 1a). An autoradiogram revealed much higher RNA levels in the UV-treated aRrp41- and aRrp4-samples (lanes 2 and 4 in Fig. 1a) when compared to the corresponding non-treated samples (lanes 1 and 3 in Fig. 1a), showing that the immunoprecipitated proteins directly interact with RNA. In CLIP experiments, the varying length of the crosslinked RNA in the immunoprecipitated RNA/Protein complex typically results in a smear above the expected molecular weight of the protein [32]. The negative control lanes (Trx, with and without UV treatment) showed similar low RNA levels (background) as the non-crosslinked aRrp41- and aRrp4-samples (compare lanes 5 and 6 to lanes 1 and 3 in Fig. 1a). A Western blot analysis of the membrane with aRrp41-specific antibodies confirmed that this exosomal subunit (and thus the exosome) was coimmunoprecipitated with the aRrp4-specific antibodies, but not with the Trx-antibodies (Fig. 1b). In summary, Fig. 1 a and b show a specific, UV-crosslink-dependent CoIP of RNA with antibodies directed against the exosomal subunits aRrp4 and aRrp41.

The iCLIP experiment was performed with *S. solfataricus* P2 cells grown to early stationary phase (Fig. 1c). Figure 1d shows the autoradiogram of the nitrocellulose membrane with all transferred, crosslinked protein-RNA complexes. The labeled areas of each lane were used for iCLIP library preparation and sequencing (see Methods). The obtained cDNA reads were mapped to the genome and used for determination of the crosslink sites (see Methods and the results below). As expected, both Trx control samples (Trx1 and Trx2) contained fewer reads than the exosome-samples (aRrp41–1, aRrp41–2, aRrp4–1 and aRrp4–2, see Additional file 2 and Fig. 1d). Mapped cDNA reads of the iCLIP data were used to determine crosslink sites representing binding sites of the exosome on its RNA substrates (Additional file 3).

### Non-mapped reads corresponding to circular RNAs

**Fig. 2:**
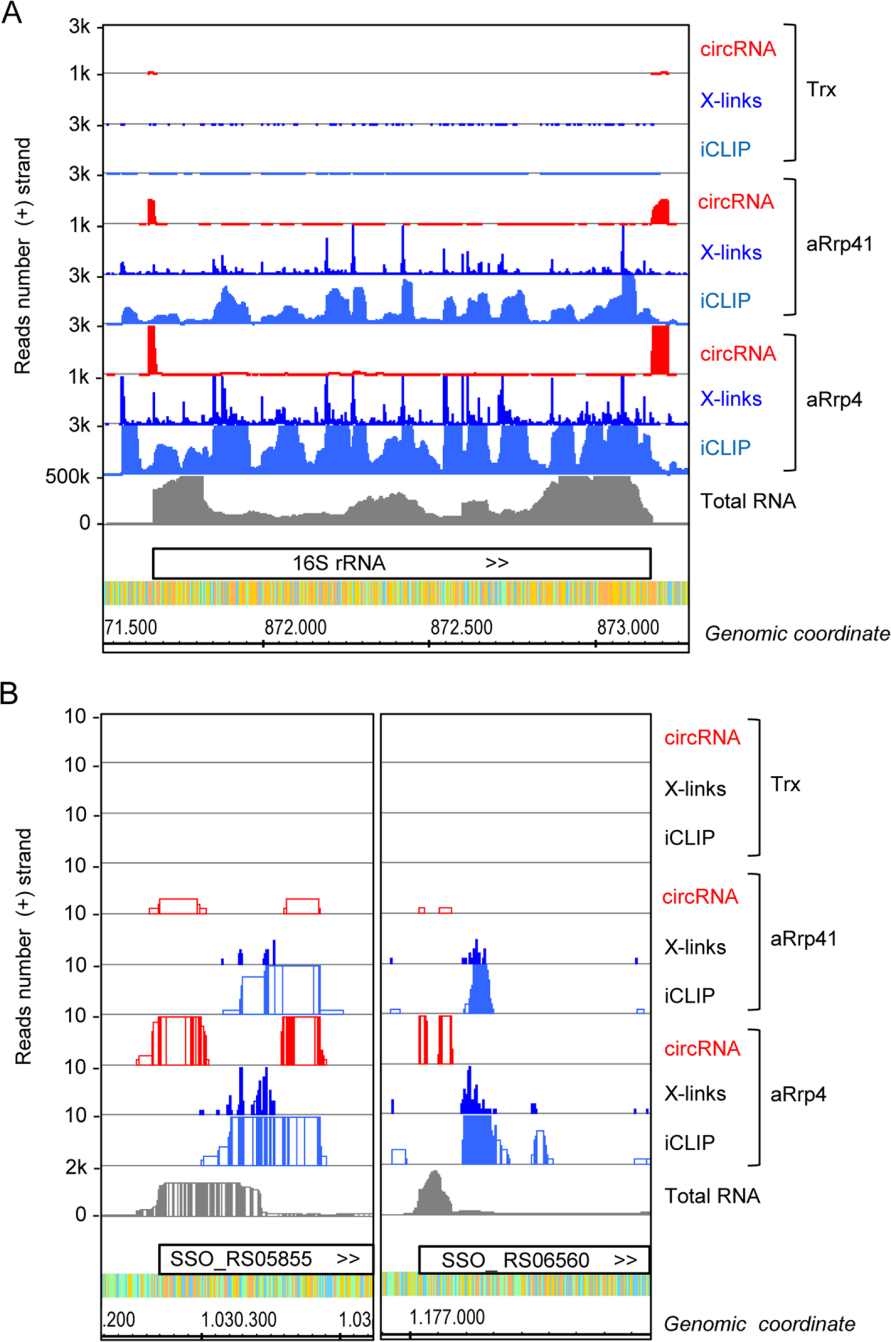
CircRNAs identified in the exosome iCLIP analysis *of S. solfataricus.* cDNA reads at the 16S rRNA locus **(a)** and at the 5′-regions of the two indicated transposase genes **(b)** are shown as coverage plots. Total RNA: cDNA reads of RNA-Seq of total RNA; iCLIP: mapped cDNA reads of the iCLIP analysis; crosslinks: corresponding mapped crosslink sites; CircRNA: cDNA reads of circRNAs detected in this study. Shown are data for aRrp41–1, aRrp4–1 and Trx-1. Annotated genes (white bars) are indicated. >>: transcript corresponding to the plus strand; <<: transcript corresponding to the minus strand. The results of the second iCLIP experiment are shown in Fig. S2 (Additional file 1)

Approximately 14 to 19% of the obtained iCLIP cDNA reads could not be mapped to the genome, in contrast to 4% in a parallel transcriptome analysis by RNA-Seq (Additional file 2). Initial evaluation of the iCLIP non-mapped reads revealed that many of them are identical. Analysis of these reads revealed that they contain two short, permuted genomic sequences that flank the 16S rRNA gene and correspond to a known circular RNA (circRNA) which is a 16S rRNA processing intermediate [38]. Figure 2a shows the 16S rRNA locus with the mapped iCLIP cDNA reads, crosslink sites and circRNA, along with RNA-Seq of total RNA as visualized by the integrated genome browser (IGB). The RNA-Seq reads were used to compare the enrichment of specific transcript segments in the iCLIP considering the relative level of the particular transcript in total RNA (see below).

A previous systematic analysis of circRNAs demonstrated that *S. solfataricus* harbors additional circularized non-coding RNAs including 23S rRNA processing intermediates, excised intron sequences from tRNA precursors, C/D-box RNAs and RNase P RNA [39]. We used a similar approach to identify circRNAs in the non-mapped reads of our iCLIP data and detected numerous circRNA candidates. However, most of them were represented by very low read numbers and were present in only one or two of the four iCLIP samples (Additional file 4). To avoid false positives, we considered only the four circRNAs with at least 10 reads in at least three of the exosome CoIP libraries (Additional file 4). The most abundant circRNA, which was coimmunoprecipitated with the archaeal exosome, corresponds to the known 16S rRNA processing intermediate mentioned above, followed by the known processing intermediates of 23S rRNA and two putative new circRNAs corresponding to the 5′-regions (the first approximately 150 nt) of two homologous transposase genes, SSO_RS05855 and SSO_RS06560 (Fig. 2b).

**Fig. 3:**
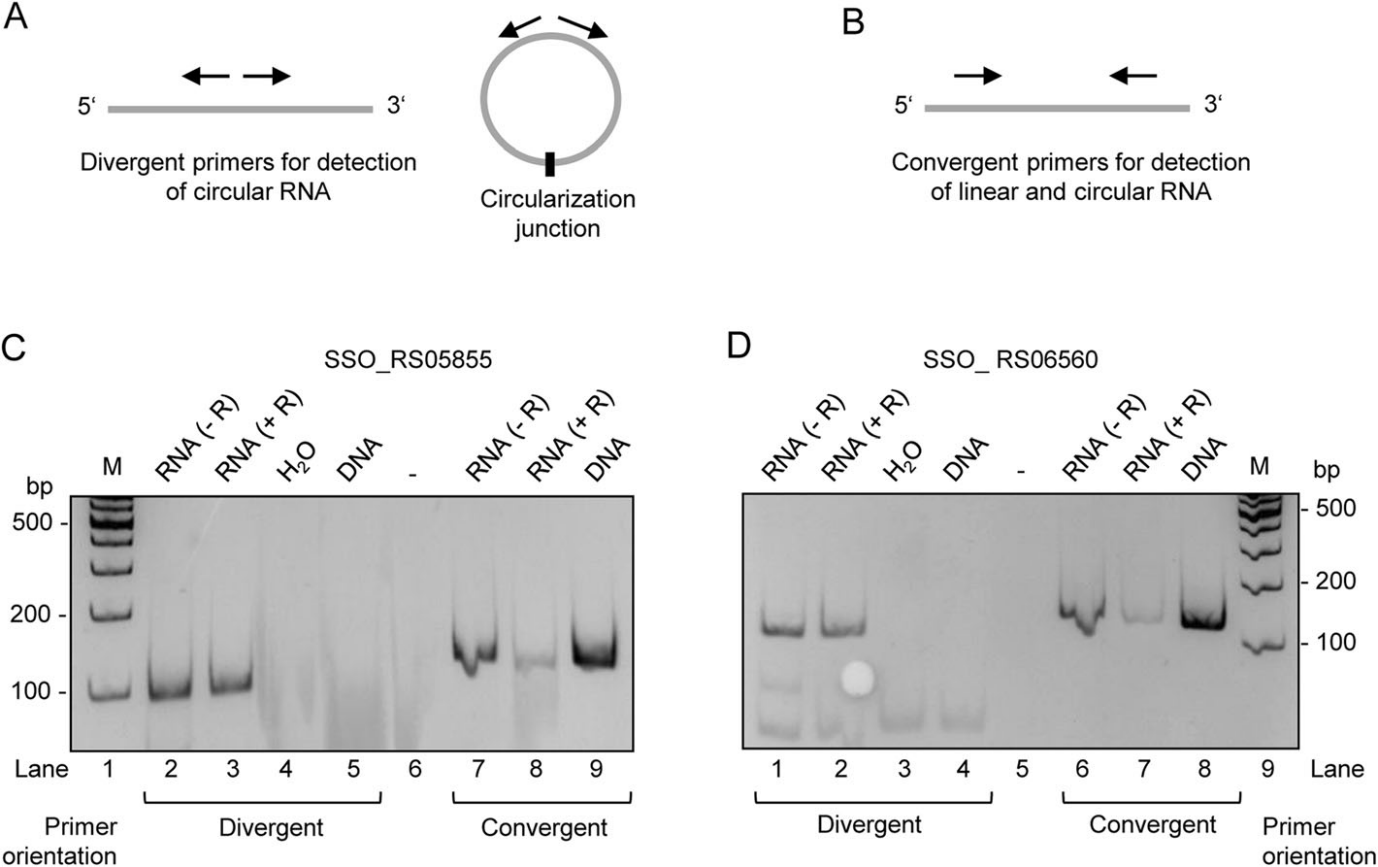
Validation of novel circRNAs corresponding to the 5′-part of the homologous transposase genes SSO_RS05855 and SSO_ RS06560. **a** and **b** Schematic representations of divergent primers for detection of circular RNA or convergent primers for detection of linear and circular RNA. Grey lines: RNA. Black arrows: oligonucleotides (primers) for RT-PCR. Short black line: circularization junctions. **c** and **d** Ethidium bromide-stained 10% polyacrylamide gels showing products from RT-PCR analyses of SSO_RS05855 and SSO_RS06560 (indicated). The use of divergent or convergent primers is given below the panels. RNA (− R): total RNA was used as template for cDNA synthesis by reverse transcriptase and then PCR was performed (RT-PCR analysis). RNA (+ R): total RNA treated with RNase R (which degrades linear but not circular RNA) prior to RT-PCR. H_2_O and DNA: positive and negative PCR control reactions, to which water or total DNA was added instead of RNA. M: length marker (size is given in bp). Uncropped images of **c**) and **d**) are shown in Fig. S1 (Additional file 1)

To validate the novel circRNAs, we performed RT-PCR analysis with RNA from an independent *S. solfataricus* culture. Circular and linear RNAs can be distinguished by treatment with bacterial exoribonuclease RNase R, which preferentially degrades linear but not circular RNA [39]. Therefore, we treated one-half of the RNA samples with RNase R, and then performed RT-PCR with the treated and non-treated RNA. Two sets of primers were used for this analysis: divergent primers suitable for detection of the circular RNA form and convergent primers for detection of both the linear and circular forms (Fig. 3a and b). As expected, when convergent primers were used, the intensity of the amplicon bands was diminished when the RNA sample was treated with RNase R (compare lane 7 to lane 8 in Fig. 3c, and lane 6 to lane 7 in Fig. 3d). In contrast, when the divergent primers for detection of circular RNAs were used, the intensity of the amplicon band was essentially not affected by the RNase treatment (compare lane 2 to lane 3 in Fig. 3c, and lane 1 to lane 2 in Fig. 3d). These results strongly suggest that the 5′-part regions of the mRNAs of genes SSO_RS05855 and SSO_RS06560 undergo circularization, and that the circularized products are interacting with the exosome.

We also attempted to detect circRNAs by existing tools. However, tools such as circRNAfinder (https://github.com/bioxfu/circRNAFinder) und CIRCexplorer [40] were not feasible, since they rely on annotation of eukaryotic splicing events. For de novo identification of circRNA candidates, we used CIRI2 [41]. The three circRNA candidates detected by this tool mapped to the 16S rRNA locus, but showed very low read numbers and were different from the above mentioned, abundant circRNA (Additional file 4). One of them was detected in two of the exosome iCLIP samples, while the other two were detected in only one of the samples (Additional file 4). This suggests that our approach based on ref. [39] is more suitable for detection of circRNAs in the exosome iCLIP samples of the archaeon *S. solfataricus* than the CIRI2 tool.

Together, the above results suggest that the exosome is involved in degradation of circRNA intermediates arising during the processing of 16S rRNA and during the degradation of transposon mRNAs.

### Non-mapped reads corresponding to A-rich RNA tails

After removal of the circRNA reads from the pool of non-mapped reads, the remaining sequences were analyzed further. We hypothesized that most of them represent posttranscriptionally added RNA tails, since such non-templated, A-rich RNAs are synthesized by the exosome and are also expected to be used by the exosome in the process of RNA degradation [21]. We decided to focus on non-templated sequences located downstream of a cDNA stretch that can be aligned to the chromosome (Additional file 5). Most of them are short poly(A) sequences, but heteropolymeric sequences were also detected. Analysis of their base content (Additional file 6) revealed that the putative RNA tails, which were coimmunoprecipitated with the exosome, contain approximately 73% adenosine.

**Fig. 4:**
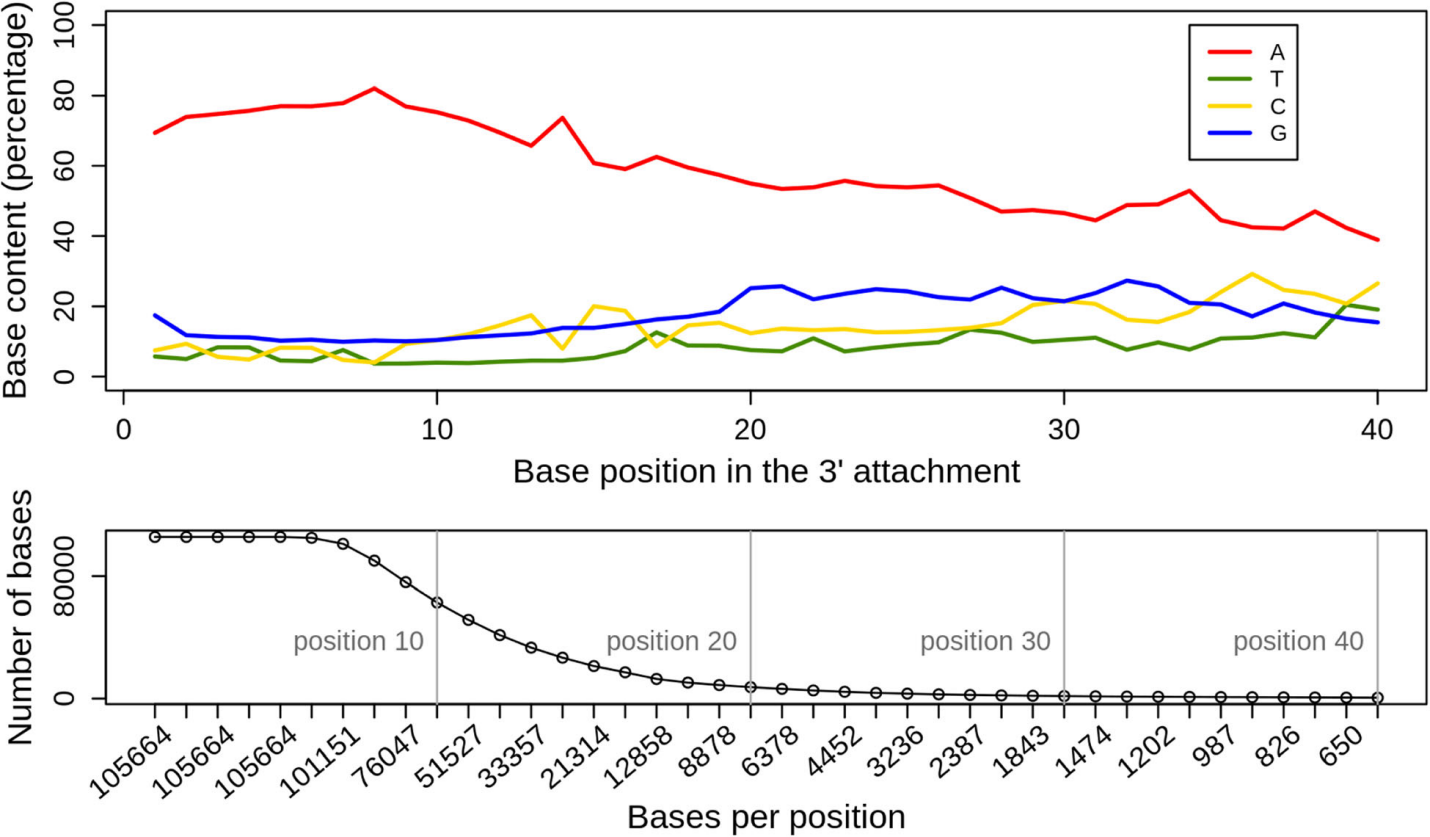
Distribution of bases in RNA-tails detected in the aRrp4-iCLIP of the archaeal exosome. The RNA tails are posttranscriptional modifications at the 3′-end (3′-attachments presumably synthesized by the exosome without a template [21]). The number of the analyzed bases per position is indicated. The maximal length of the analyzed tails was set to 40 nt

We further analyzed the nucleotide composition for each position in the coimmunoprecipitated, putative RNA-tails and found that the first 10 attached nucleotides were mostly A (> 70%), and A was clearly prevalent also in longer tails (Fig. 4 and Fig. S3 in Additional file 1). This is in line with the exosome function in synthesizing A-rich RNA-tails [21] and with its preference for binding of poly(A) [10, 11]. The very short length of the most detected RNA-tails (see the bottom panels in Fig. 4 and Fig. S3) is in line with previous results [21].

Based on the above results we suggest that most of the here detected, non-mapped, poly(A) and A-rich reads (Additional file 5) represent natural, posttranscriptionally added RNA-tails, which were in close physical interaction with the exosome in *S. solfataricus*.

### Poly(A) stretches in chromosomally templated RNA bound by the archaeal exosome

**Fig. 5:**
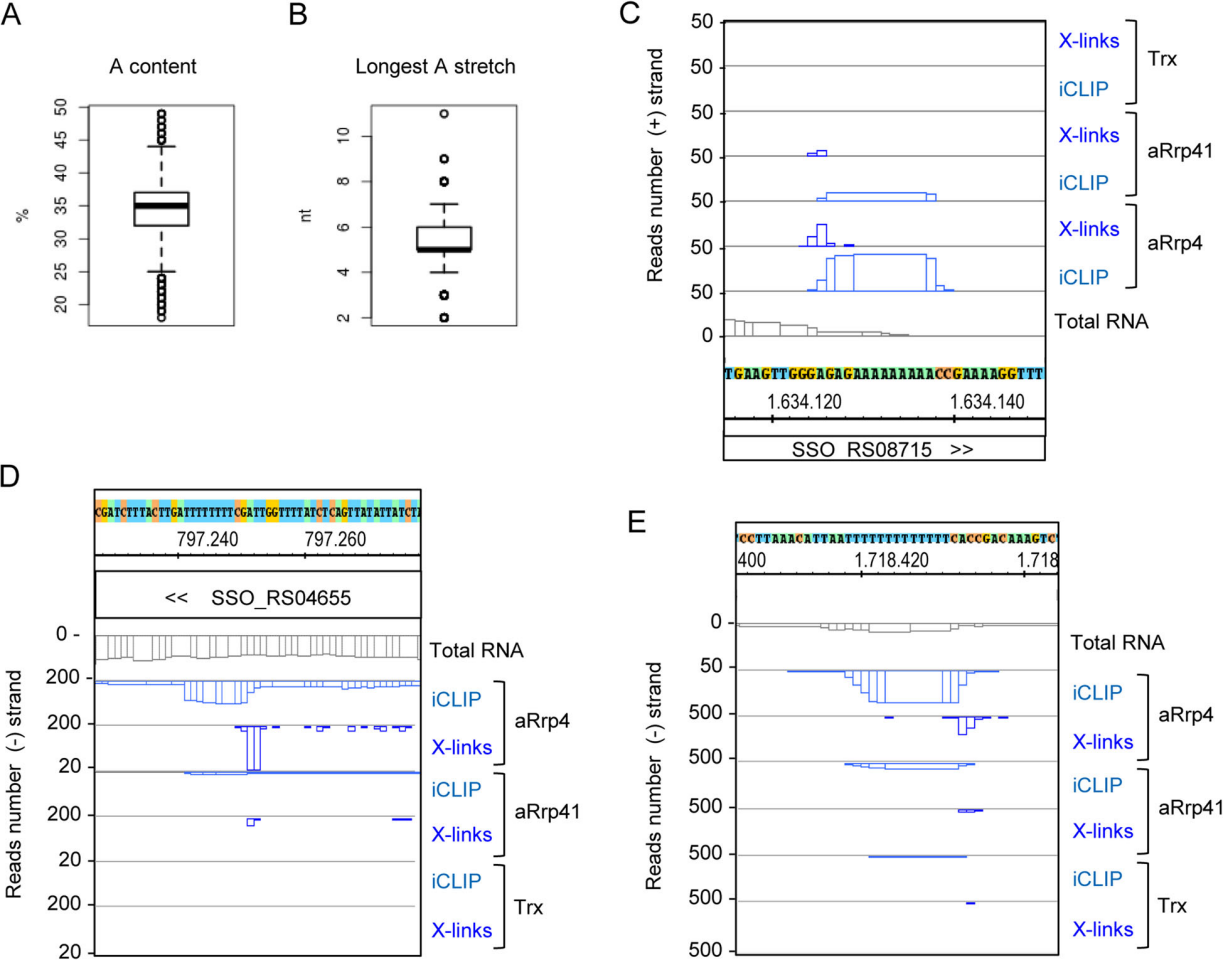
Poly(A) stretches in RNA are enriched in the iCLIP. **a** Boxplot of the relative A-content in all 2977 annotated *S. solfataricus* CDS. **b** Boxplot of the longest poly(A) stretch for each of the 2977 annotated *S. solfataricus* CDS. **c** cDNA reads and crosslink sites in the SSO_RS08715 CDS encoding a transposase. **d** cDNA reads and crosslink sites in the SSO_RS04655 CDS encoding glutamyl-tRNA (Gln) amidotransferase subunit D. **e** cDNA reads and crosslink sites in the intergenic region between the convergently transcribed genes SSO_RS09125 (on the plus strand, encodes a hypothetical protein) and SSO_RS09130 (on the minus strand, encodes energy-coupling factor transporter transmembrane protein EcfT). For other descriptions see Fig. 2. The results of the second iCLIP experiment are shown in Fig. S4 (Additional file 1)

The poly(A) preference of two of the RNA binding proteins of the archaeal exosome [10, 11] and previous detection of purine-rich RNA-tails in *S. solfataricus* [21] suggested that A- and AG-rich mRNAs might be preferred substrates of the archaeal exosome. To address this, the A- and AG-content of annotated protein-coding genes (CDS) of *S. solfataricus* was analyzed (Additional file 7). The A- and AG-content results were similar and below we briefly describe the A-content results. Most CDS of *S. solfataricus* have an A-content between 32 and 37% (Fig. 5a). The lowest A-content (18%) was found in transposon ISC1395 ORFs, while genes encoding ribosomal proteins L29, L31 and S17, which belong to an operon, have the highest A-content of 49%. However, these ribosomal mRNAs were not enriched in the iCLIP (lower numbers of iCLIP cDNA reads were obtained when compared to RNA-Seq of total RNA), probably because they are highly translated.

We also considered the possibility that poly(A) stretches in transcripts may contribute to their recruitment by the poly(A)-preferring subunits of the exosome and analyzed the length of continuous (A)_n_ sequences in coding sequences (CDS). A median of 5 nt poly(A) was observed, with 22 CDS above the 7 nt upper quantile (Fig. 5b and Additional file 7). Besides a number of transposon ORFs and hypothetical proteins, this group with relatively long poly(A) stretches contains the alanyl-tRNA synthetase, signal recognition particle receptor, glutamyl-tRNA (Gln) amidotransferase subunit D and the quinol oxidase CDS (SoxABC/soxC). Among the 22 CDS, six transposon ORFs belong to repetitive sequences, which were not used in our iCLIP analysis. The poly(A) stretches of the remaining 16 ORFs were examined for enrichment in the iCLIP. As enrichment we considered at least 2-fold more reads in the iCLIP than in the RNA-Seq, in at least one of the iCLIP samples. This enrichment definition was supported by the genome-wide analysis of the ratio of iCLIP to RNA-Seq coverage for each CDS position, the median of which was below 0.06 and the third quartile below 0.20 (Fig. S4 in Additional file 1). Such an enrichment was detected in three CDSs harbouring (A)_11_, (A)_9_ and (A)_8_, respectively (Additional file 8). An example is the transposon ISC1048 ORF1 mRNA that contains (A)_9_ (Fig. 5c). Furthermore, peaks of iCLIP reads were observed at the poly(A) stretches of five of the remaining 13 CDS, suggesting exosome binding at the poly(A) region of the mRNAs (Additional file 8). An example is shown in Fig. 5d, at the (A)_8_ region of SSO_RS04655 mRNA encoding glutamyl-tRNA (Gln) amidotransferase subunit D.

We also analyzed poly(A) stretches in intergenic regions (IGRs) that may correspond to 5′- and 3′-UTRs or non-coding RNAs. These poly(A) stretches were identified using blastn with (A)_30_, and the top 20 hits were analyzed (Additional file 8). At one of them, no reads were mapped in the RNA-Seq and in the iCLIP analyses, suggesting that this genomic region is not transcribed under the used conditions. At the poly(A) stretches of 13 of the remaining 19 IGRs, enrichment in the iCLIP was detected. The enrichment was defined as at least 2-fold higher peak in iCLIP compared to RNA-Seq, or a peak in iCLIP although no RNA-Seq reads were mapped, in at least one of the samples. A genome-wide analysis of the ratio of iCLIP reads to RNA-Seq reads at each IGR position in the four exosome iCLIP samples revealed median values of maximally 0.06 and 75% quartile values of maximally 0.33 (Fig. S4 in Additional file 1). As an example, Fig. 5d shows the aRrp4 iCLIP enrichment of an (A)_13_-containing RNA that most probably corresponds to a 3′-UTR. This enrichment suggests strong or preferential RNA binding by the archaeal exosome.

Although Fig. 5, which shows results of the first iCLIP replicate, suggests that RNA with poly(A) stretches was coimmunoprecipitated mainly when aRrp4-directed antibodies were used, the second iCLIP replicate revealed that such RNA was coimmunoprecipitated by both aRrp4- and aRrp41-directed antibodies (Fig. S4 in Additional file 1).

### Identification of mapped RNAs preferentially bound to the exosome

The iCLIP method was developed for identification of distinct sets of RNA binding sites at high-resolution [32]. The archaeal exosome is expected to bind to many different RNAs, and, due to its processive exoribonucleolytic activity, to occupy many different positions in a particular substrate by successively shortening it from the 3′-end. Despite this, it can be expected that some cellular RNAs are bound and processed or degraded with higher preference. Alternatively, some substrates may occupy the exosome for a longer time if they are degraded more slowly than others are. Such substrates should be enriched after UV crosslinking and immunoprecipitation with exosome-specific antibodies.

To identify by iCLIP RNAs that are preferentially bound to the exosome (enriched RNAs), we compared the read numbers obtained in the iCLIP experiments with the read numbers from an RNA-Seq analysis of total RNA isolated from *S. solfatraricus* that was grown under the same conditions (TPM normalization of RNA-Seq read numbers was applied; see Fig. S5 in Additional file 1 and Additional file 9). Particularly, tRNAs were preferentially bound by the exosome. Few examples of enriched transcripts and transcript parts are given below. Additionally, iCLIP and RNA-Seq reads coverage of random regions of the *S. solfatarics* genome is shown in Fig. S5 (Additional file 1).

**Fig. 6:**
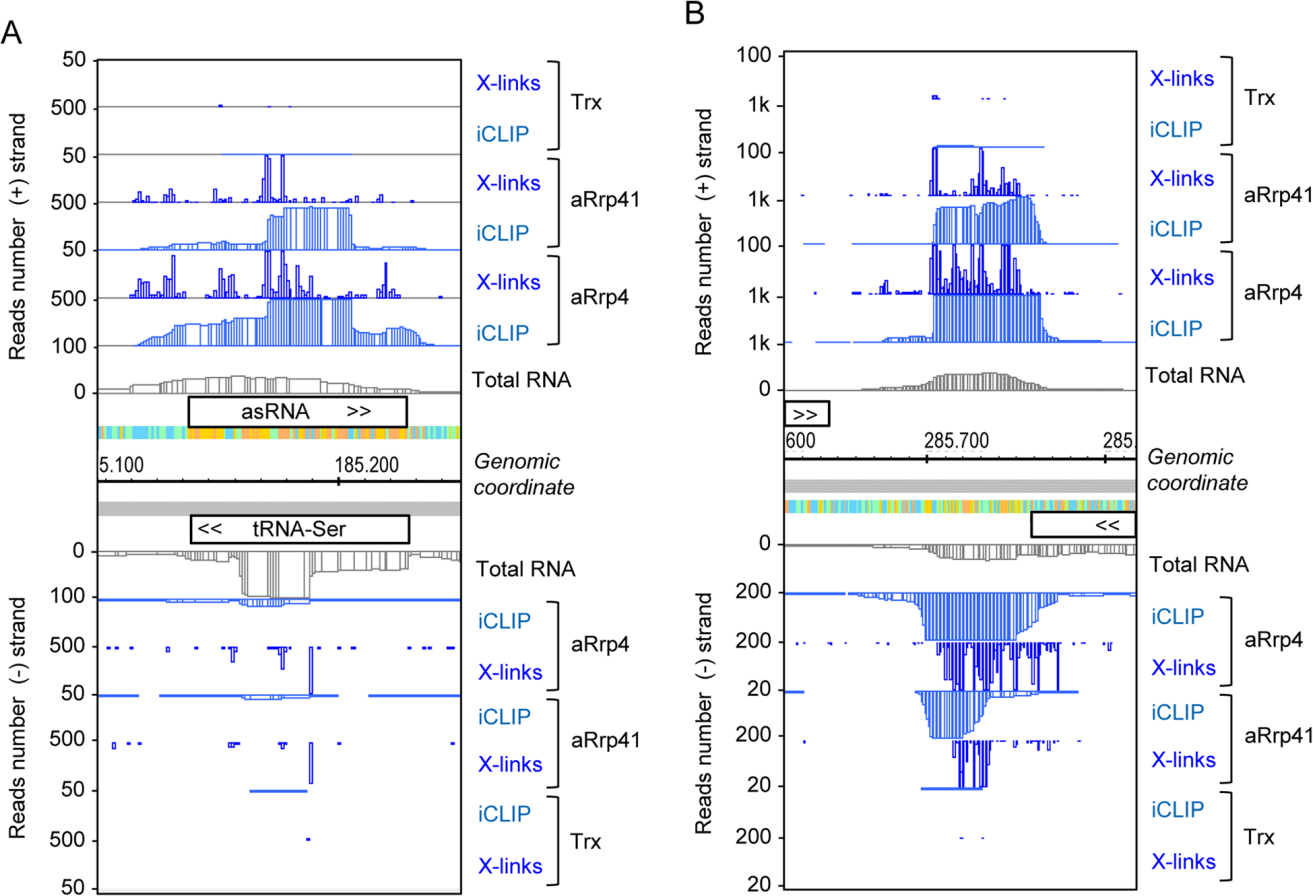
Antisense RNAs as preferred substrates of the archaeal exosome. **a** cDNA reads and crosslink sites at the tRNA-Ser locus (SSO_RS01080, alias SSOt12) show strong enrichment of antisense RNA in the iCLIP. **b** cDNA reads and crosslink sites in the intergenic region between two convergently transcribed genes (SSO_RS01650 encoding a hypothetical protein on the plus strand and SSO_RS01655 encoding tRNA 4-thiouridine (8) synthase ThiI on the minus strand) show strong iCLIP enrichment of potentially antisense transcripts corresponding to overlapping 3′-UTRs. For other descriptions see Fig. 2. The results of the second iCLIP experiment are shown in Fig. S6 (Additional file 1)

A prominently enriched transcript was an antisense RNA (asRNA) that is complementary to tRNA-Ser (Fig. 6a). RNAs that are antisense to tRNAs are common in *S. solfataricus*, but their function remained unclear [38]. According to our data, such asRNAs are preferred substrates of the exosome, suggesting that the exosome may be involved in the clearance of asRNAs.

We also observed enrichment of antisense RNAs together with their complementary counterparts, which presumably correspond to overlapping 3′-UTRs of convergently transcribed genes (Fig. 6b). The enrichment of RNAs from both strands suggests that the exosome participates in the degradation of double-stranded RNA (dsRNA). Alternatively or in addition, dsRNA may occupy the exosome due to its slower degradation in comparison to single-stranded RNA (ssRNA). Interaction of the exosome with asRNAs may also point to a role of the asRNA in promoting an exosome-mediated mRNA degradation.

**Fig. 7:**
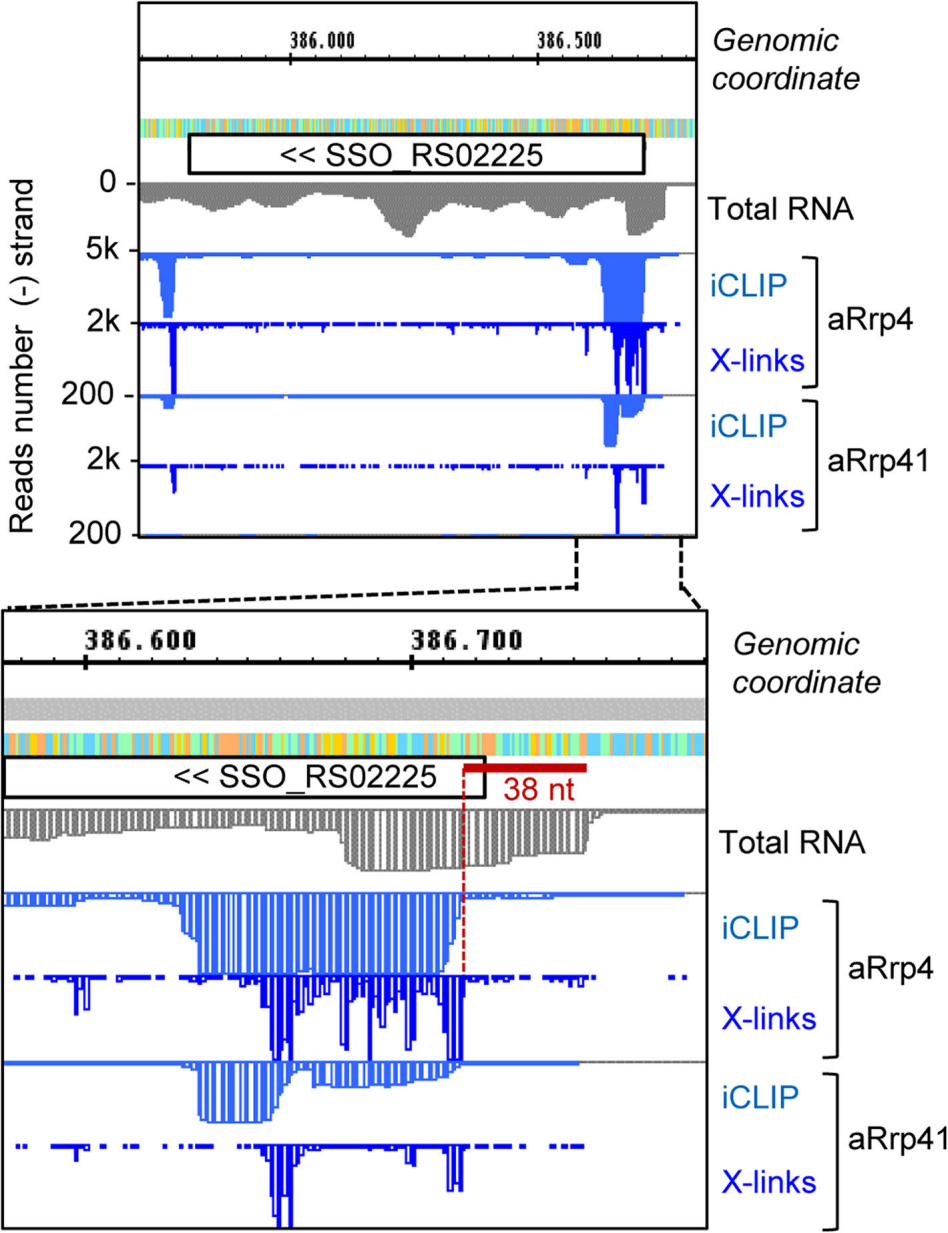
Binding of the archaeal exosome to the 5′-part and the 3′-UTR of the *tfb* gene. The gene *tfb* (SSO_RS02225) encodes transcription factor TFIIB cyclin-related protein. Shown are cDNA reads and crosslink sites of the SSO_RS02225 locus and the flanking regions (top panel), and a zoom in the 5′-part of the gene (bottom panel). The first 38 nt of the transcript including the 5′-UTR and the first codons were not crosslinked (marked with a red line). For other descriptions see Fig. 2. The results of the second iCLIP experiment are shown in Fig. S7-C (Additional file 1)

Interestingly, we observed crosslink sites clustering near start and stop codons of CDS (for examples, see Fig. 7 and Fig. S7 in Additional file 1; see also Fig. 9 below). Figure 7 shows *tfb* (SSO_RS02225) encoding transcription factor TFIIB cyclin-related protein and its flanking regions. Clustering of crosslink sites was detected near the start of the *tfb* ORF and downstream of the ORF, in the presumable 3′-UTR (Fig. 7, upper panel). The RNA-Seq data suggested that the 5′-UTR of *tfb* is 30 nt long and the iCLIP analysis revealed that the first 38 nt of the detected transcript including the 5′-UTR and the first codons were essentially not crosslinked (marked with red lines in Fig. 7, bottom panel). Instead, the data suggest binding of the exosome to the region + 40 to + 120 of *tfb* mRNA (+ 1 being the presumable transcription start site).

It is noteworthy that the highly abundant rRNAs were not enriched by the exosome (see Fig. 2a above), strongly suggesting that the above-described enrichment reflects the preferential occupancy of the exosome by specific RNA substrates. Among the coimmunoprecipitated sequences were rRNA flanking regions as well as internal rRNA regions (see Fig. 2a above). The mapped crosslink sites probably represent 1) binding of the exosome to rRNA precursors during maturation of rRNA and 2) degradation of rRNA that serves to remove non-properly maturated or damaged rRNA. As additional example for an RNA that was not enriched by the iCLIP we show the highly abundant *tmoA* mRNA (Fig. S8 in Additional file 1). This underlines the specificity of our iCLIP results. For iCLIP and RNA-Seq reads coverage of random genomic regions, see Fig. S5 in Additional file 1.

### Most crosslink sites and read counts correspond to mRNAs

**Fig. 8:**
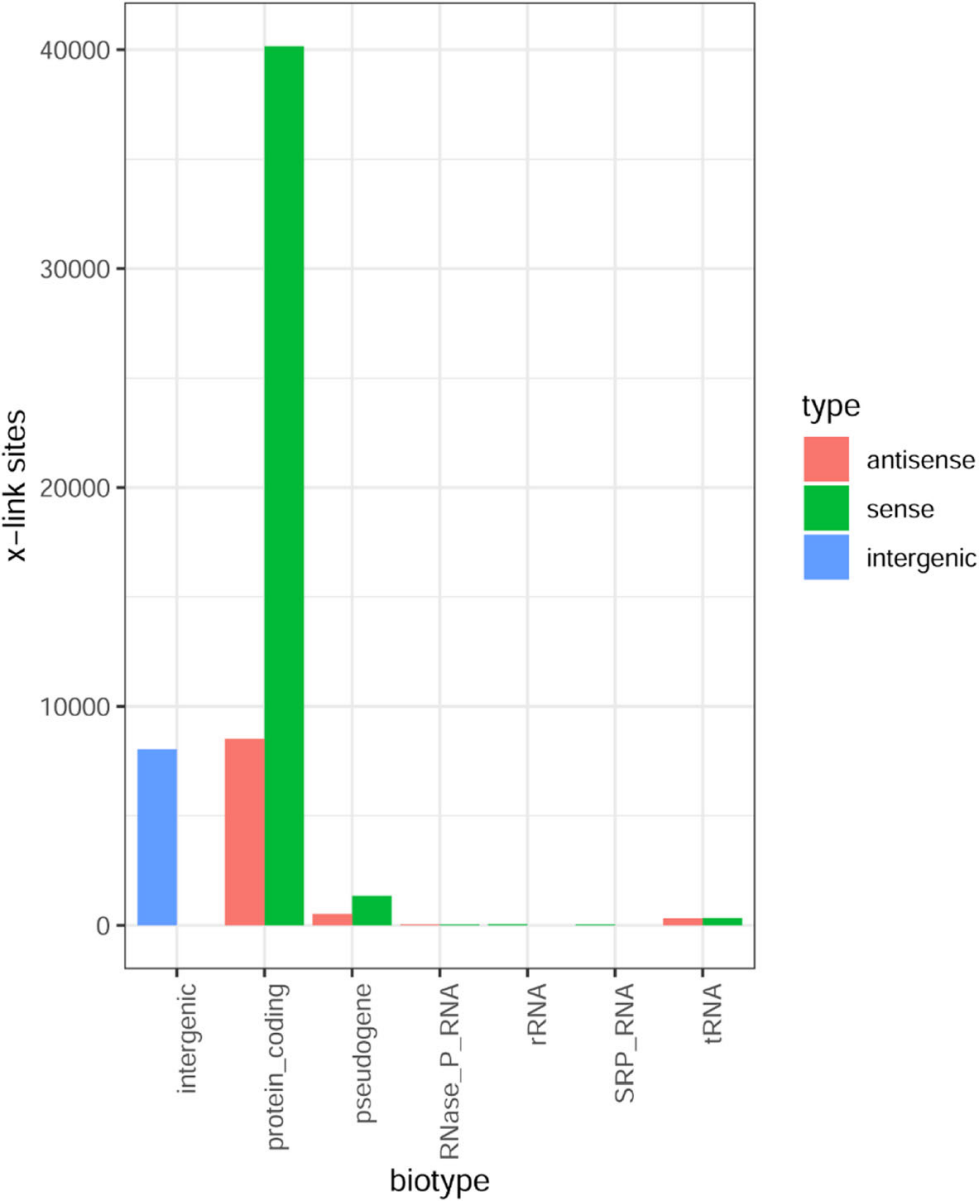
Detection of most crosslink sites on the sense strand of protein coding genes. Distribution of crosslink sites in the genome of *S. solfataricus* was analyzed with respect to biotype RNA

Assuming that each cDNA read corresponds to an exosomal complex bound to an RNA molecule in the cell, the iCLIP data offer a possibility to determine which RNA classes occupy the exosomes in *S. solfataricus*. To address this, the transcriptome-wide distribution of crosslink sites was analyzed. However, due to the exoribonucleolytic nature of the exosome no clear individual peaks were obtained in the iCLIP (see Fig. 6, Fig. 7 and Fig. S6 and Fig. S7 in Additional file 1). Thus, we considered the possibility that crosslink sites were assigned to most prominent 5′-end peaks and many exosome binding positions represented by cDNA reads could be missed. Therefore, in addition to the distribution of crosslink sites, the transcriptome-wide distribution of read counts was analyzed. According to Fig. 8, the vast majority of crosslink sites was mapped in mRNAs (sense strand of protein-coding genes), followed by intergenic regions and asRNAs. Similarly, the vast majority of read counts was mapped in mRNAs (Fig. S9 in Additional file 1), suggesting that the crosslink sites results are not strongly biased. In summary, most exosomal complexes in the cell are occupied by mRNAs.

### Unexpected clustering of crosslink sites near start of protein coding genes

**Fig. 9:**
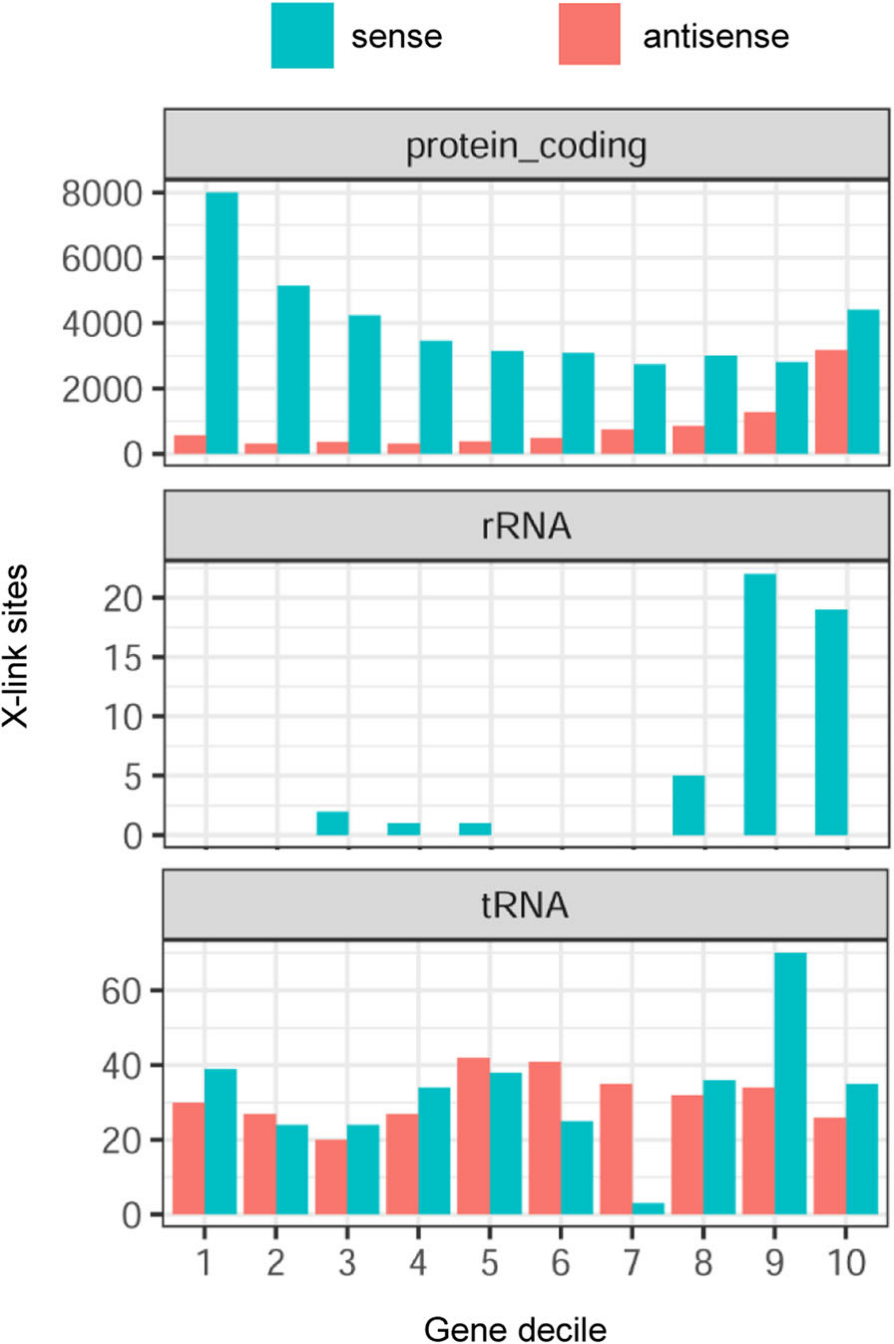
Clustering of crosslink sites at the 5′- and 3′-end of protein coding genes. The crosslink sites per decile of annotated genes was analyzed. Gene biotype and transcript orientation are indicated

As a consequence of the results presented in Fig. 7 (see also Fig. S7), we analyzed the distribution of crosslink sites in annotated genes and found strong enrichment in the first, 5′-end decile of protein-coding genes (Fig. 9, Fig. S10 in Additional file 1) and predicted operons (Fig. S11 in Additional file 1). The relative number of crosslink sites decreased in 5′ to 3′ direction, and increased again in the last, 3′-end decile. The crosslink sites peak in corresponding asRNA was in the last gene decile (thus, possibly near the 5′-end of the asRNA). In contrast, in rRNA genes most crosslink sites were located in the last two gene deciles, while in tRNAs and their asRNAs the crosslink sites were distributed more evenly (an exception were the 7th and 9th tRNA deciles) (Fig. 9). Similar results were obtained by the analysis of read counts (Fig. S10 in Additional file 1).

**Fig. 10:**
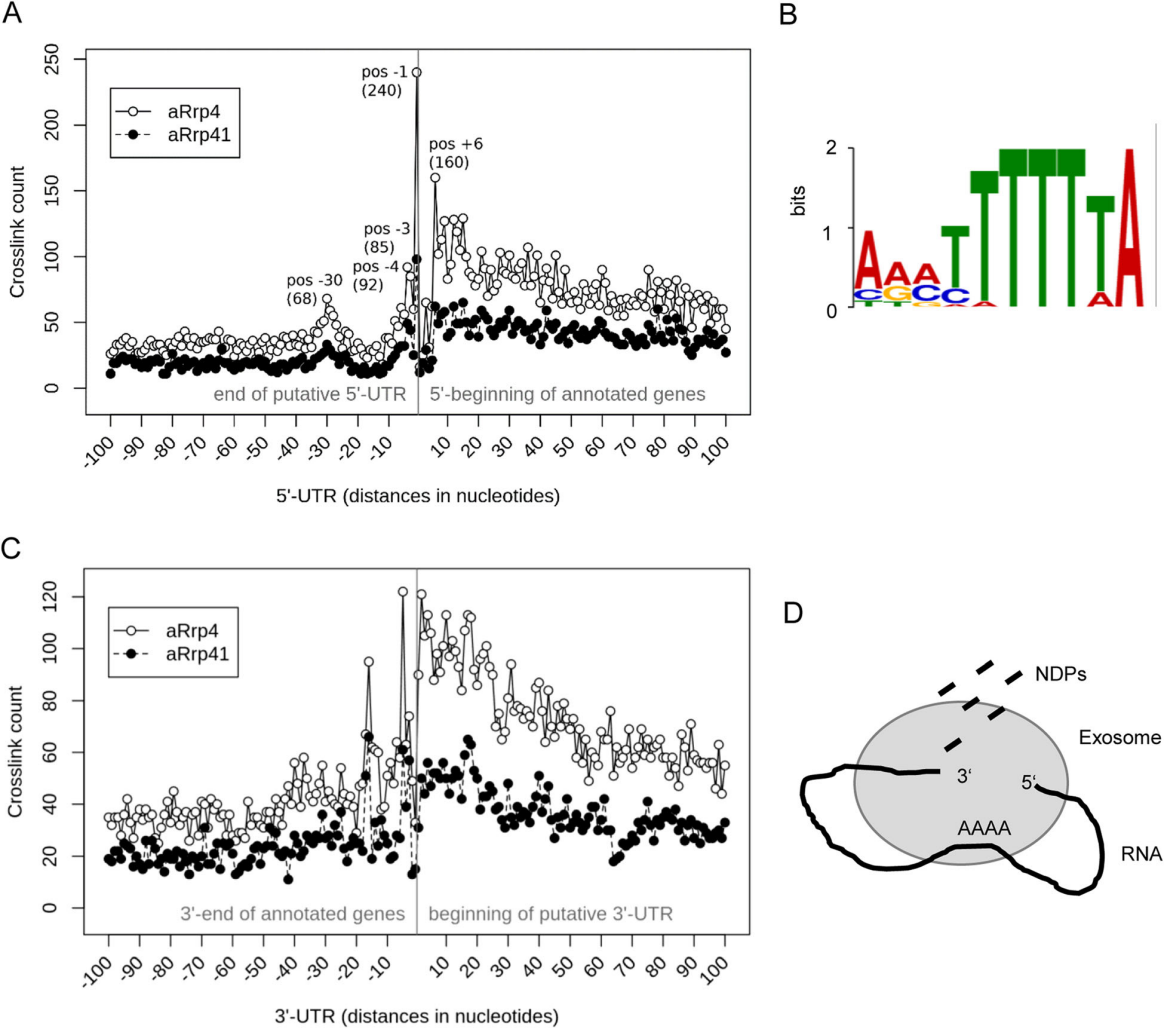
Distribution of crosslink sites in annotated genes and their UTRs. **a** Positions of crosslink sites around the start of annotated genes. Negative nt positions correspond to 5′-UTRs, positive positions are located in the genes. One hundred nt upstream and downstream of the gene starts were analyzed. **b** Logo of the sequence motif that was detected by meme for the peak at position − 30 in the 5‘-UTR. **c** Positions of crosslink sites around the end of annotated genes. Negative nt positions are located in the genes, positive positions correspond to the 3’-UTRs. One hundred nt upstream and downstream of the gene ends were analyzed. **d** Model for circular degradation of RNA by the archaeal exosome. RNA is degraded exoribonucleolytically in 3′-5′-direction in a processive manner, and nucleoside diphosphates (NDPs) are released. During the degradation process, the 5′-end of the RNA substrate is bound to the exosome. In the iCLIP analysis, this results in an even distribution of crosslink sites in the RNA body and clusters of crosslink sites in the 5′-region. Preferential binding of poly(A) stretches is indicated

Next, we addressed the question whether the higher number of crosslink sites at ends of protein coding genes is due to preferential binding of the exosome to 5′- and 3′-UTRs (or 5′-leaders and 3′-trailers). To answer this question, the distribution of crosslink sites in a 100 nt window centered around the 5′ and 3′ ends of annotated genes was analyzed (Fig. 10a and b; see also Fig. S11 and Fig. S12).

Figure 10a shows the distribution of crosslink sites around the 5′-end of genes. In the 5′-UTRs, a high peak was detected at position − 1, followed by peaks at positions − 3, − 4 and − 30. In the reads corresponding to the − 30 peak, an AAATTTTTTA-motif was found (Fig. 10b). The physiological role of this motif is not clear. Generally, more crosslink sites were detected at position + 6 and downstream in the genes than in the 5′-UTRs (Fig. 10a). However, a strikingly low number of peaks was detected between position + 1 and position + 6 in the genes. This “peak gap” may correspond to oligoribonucleotides that arise as products of exoribonucleolytic degradation of leaderless mRNAs that are prevalent in *S. solfataricus* [42]. The peaks just upstream of the start codon may represent 5′-ends of such leaderless mRNAs.

The distribution of crosslink sites around the 3′-end of annotated genes is shown in Fig. 10c. Generally, more crosslink sites were detected in the 3′-UTRs than in the genes, with the highest peak directly downstream of the 3′-end of the genes. This suggests that translation protects mRNA from degradation by the exosome. In the case of non-coding RNAs, this result may reflect 3′-end trimming by the exosome. Additionally, two high peaks were detected 5 and 18 nt upstream of the 3′-end of genes were detected, for which we do not have an explanation.

## Discussion

To the best of our knowledge, this is the first iCLIP study of an exoribonuclease. Our results highlight the role of the archaeal exosome as a major RNA processing and RNA degrading enzyme, which interacts with all classes of RNAs. Particularly, they suggest a new model of mRNA degradation (Fig. 10d).

The presence of many cDNA reads that could not be mapped to the genome was not surprising, because binding of the exosome to heteropolymeric, A-rich tails that are synthesized by the exosome [21], was expected. However, in addition to the A-rich tails, circRNAs were prominent among the non-mapped reads. In addition to the known circRNAs representing 16S and 23S rRNA processing intermediates [38, 39], new circRNAs corresponding to 5′-portions of transposase mRNAs were detected in our iCLIP analysis. These new circRNAs were not detected in the high-throughput study by Danan et al. [39], who did not validate mRNA-derived circRNAs. In our study, the validated SSO_RS05855- and SSO_RS06560-derived circRNAs were among the most abundant circRNAs that were coimmunoprecipitated with the exosome, and were even more abundant than tRNA-derived introns. This suggests that these circRNAs are specifically interacting with the exosome. In the cell, this could contribute to their clearance by the exosome, if an endoribonuclease converts the circular RNA to a linear form that is accessible for exoribonuceolytic degradation [3]. Alternatively, degradation may also be started by spontaneous linearization under the harsh condition to which *S. solfataricus* is exposed due to its lifestyle. Indeed, Danan et al. [39] proposed that RNA circularization might represent a part of the RNA degradation pathway for some RNAs. Since SSO_RS05855- and SSO_RS06560 encode transposases, our results suggest that the exosome participates in the posttranscriptional regulation of transposition and genome rearrangements, which are prevalent in *S. solfataricus* [43].

Based on the poly(A) preference of the aRrp4 and aDnaG subunits of the exosome, we expected preferential interaction of the protein complex with the most A-rich mRNAs. However, our results suggest that not the higher A-content of certain mRNAs, but rather the presence of internal short poly(A) sequences is a determinant for RNA recruitment by the archaeal exosome. Comparison of the data indicating exosome binding to poly(A) stretches in CDS or IGRs (Additional file 8) suggests strong and/or preferential exosome binding at poly(A) stretches in non-coding regions. This could be explained by better accessibility of non-coding RNA regions: ribosome coverage of the poly(A) stretches in CDS probably prevents exosome binding at those positions. Further analyses of RNAs enriched by the exosome suggested that in the cell, the exosome is strongly associated or binds for a longer time to tRNAs (Fig. S5 in Additional file 1), specific antisense RNAs, and specific transcript parts as 3′-UTRs and 5′-UTRs.

The most crosslink sites and most read counts were mapped to mRNA, although rRNA and tRNA is present in much higher amounts in the cell. This could be explained by the applied conditions, since it can be expected that in the early stationary phase (Fig. 1a) rRNA and tRNA genes are not strongly transcribed and therefore less such precursor transcripts are processed or degraded. Furthermore, in the early stationary phase mature rRNA and tRNA is probably not degraded. This result supports the view that mRNA turnover is a major function of the archaeal exosome. Messenger RNAs are instable in S. *solfataricus* and generally in prokaryotes, a feature that is important for adaptation to rapidly changing environmental conditions [44, 45].

The analysis of crosslink sites distribution in genes and their UTRs led to interesting results. While preferential detection of crosslink sites in 3′-UTRs and 3′-trailers was expected due to the function of the exosome as a 3′-5′ exoribonuclease, the clustering at the 5′-end of annotated genes was surprising. One possible explanation for the 5′ clustering is an interaction of the 5′-end of mRNAs with the exosome. This suggests that while the exosome degrades or processes RNA at the 3′-end, it also binds to the 5′-end and thereby “circularizes” the transcript (Fig. 10d). The physical proximity of 5′ and 3′ parts of exosome-bound transcripts may enhance RNA circularization, for example by promoting formation of secondary structures with bulge-helix-bulge and bulge-helix-loop motifs [46]. Previously, it was proposed that RNA circularization might generally be a part of RNA degradation in *Sulfolobus* [39]. Furthermore, recently it was shown that in Euryarchaeota the 5′-3′ degrading exoribonuclease aRNase J interacts with aCsl4 and is in a complex with the exosome [47]. This leads to simultaneous binding of 5′ and 3′ transcript ends in a similar manner as proposed in Fig. 10d for *S. solfataricus*, which belongs to the Crenarchaeota. However, in *S. solfataricus* aRNase J was not found in a stable complex with the exosome [36, 48]. Thus, either the exosome itself interacts with the 5′-end of transcripts, or an exosome interacting partner that binds RNA 5′-ends remains to be identified in *S. solfataricus*.

## Conclusions

In this work, we present an iCLIP analysis of an exoribonuclease. In line with the role of the archaeal exosome as a major 3′-5′ exoribonuclease and a polynucleotidylation enzyme, the mapped crosslink sites were distributed along mRNAs and antisense RNAs, clustered in 3′-trailers and were also detected in non-templated 3′-tails. Our data strongly suggest that the majority of the exosomal complexes in the cell are degrading mRNA molecules, in line with the high instability of prokaryotic mRNA and the importance of this instability in adaptation to environmental changes. Additionally, a new insight into the mechanism of RNA degradation in archaea was obtained by the surprising clustering of crosslink sites in the first decile of protein coding genes. This observation suggests simultaneous binding of 5′- and 3′-ends by the exoribonucleolytic exosome and may serve to prevent translation of mRNAs dedicated to degradation in 3′-5′ direction.

## Methods

### Cultivation and harvesting of S. solfataricus

*S. solfataricus* P2 was grown as previously described in a 10 l bioreactor [49]. Cells were harvested by centrifugation at 6000 g and 4 °C for 10 min.

### Isolation of RNA and RT-PCR

Total RNA was isolated with TRIzol and residual DNA was digested with TIRBO-DNase. The RNase samples were divided into two halves, and one of them was treated twice with RNase R. Circular RNAs were validated as described by Danan et al. [39]. Following oligonucleotides (primers) were used for reverse transcription followed by semiquantitative PCR. The divergent primers GTTAAAAGCCCAGTTGAAGTTAAC and CCCTAAGGGTTTGTTCGTCAG were used for detection of the circular RNA derived from SSO_RS05855 mRNA, while the convergent primers AATGCCCACCTTAGGGTTTCG and GTCTGCCCACCTTAAGGTGTTG were used for the detection of the linear form of the corresponding part of SSO_RS05855 mRNA. Similarly, the divergent primers AAAGGCCCAGTTGAAGTTAGCG and AACGCCCTAAGGGTTTGTTCG were used for detection of the circular RNA derived from SSO_RS06560 mRNA, and the convergent primers TGCCCACCTTAGGGTTTCGCTTC and ATGTCTGCCCACCTTAAGGTG were used for the detection of the corresponding linear form of SSO_ RS06560 mRNA. Since the analysed mRNA regions of both genes are very similar to each other and to additional 11 loci in the genome of *S. solfataricus* P2 (the sequences of the 13 loci share at least 91% identity), we took care that one of the primers for detection of circular RNAs targets an unique sequence, ensuring the specific detection of circular RNAs. For the RT-PCR (25 cycles of cDNA amplification), Brilliant III Ultra-Fast SYBR® Green QPCR Master Mix (Agilent Technologies) was used. Each 10 μl reaction mixture contained 100 ng RNA. The PCR products were separated in 10% polyacrylamide gels with TBE buffer and stained with ethidium bromide.

### iCLIP analysis and RNA-Seq

Individual-nucleotide resolution crosslinking and immunoprecipitation (iCLIP) analyses were performed as described in [32], with the following alteration that were necessary for the application in *S. solfataricus*. Cells were cultivated until an OD_600_ of 0.7 and harvested as described above. Cell pellets were washed with PBS, 0.8 g were resuspended in 40 ml PBS at 4 °C, and spread on a large petri dish swimming on an ice-water bath. The cell suspension was crosslinked four times at 254 nm, 300 mJ/cm^2^, cells were mixed in between. Crosslinked cells were harvested as before and stored at − 80 °C.

For cell lysis, 0.5 g of cells were thawed on ice, resuspended in lysis buffer (10 mM Tris pH 7.5, 150 mM NaCl, 5 mM MgCl2, 10% glycerol, 0.1% Nonidet P40, 1 mM PMSF) and lysed by sonication (five 30 s cycles, 70%). Cells debris was removed by centrifugation, and supernatant was DNase- and RNase-treated. 10x RQ1 DNase buffer was added to 1x final concentration, and 1:500 vol. Turbo-DNase (Ambion), 1:1000 vol. RNaseOUT (Thermo Fisher Scientific) and different dilutions of RNase I (Ambion), in RQ1-buffer (high RNase: 1:1000 vol. and low RNase 1:10,000 vol.). Extracts were incubated for 6 min at 37 °C in a shaking water bath. All following steps were performed on ice / at 4 °C.

Immunoprecipitation was performed as described in [20]: 4.5 ml of lysate was incubated with Protein-A-Sepharose beads coupled to 100 μl of polyclonal antibody raised against His-tagged aRrp41 or aRrp4 [36], or polyclonal antibody raised against Thioredoxin (Trx) from the alphaproteobacterium *R. sphaeroides* [37] as a negative control, for 2 h. Beads were washed 10 times with high salt wash buffer (10 mM Tris pH 7.5, 1 M NaCl, 5 mM MgCl2, 10% glycerol, 0.1% Nonidet P40, 1 mM PMSF). To remove excess of salt, beads were then washed two times with PNK-buffer (70 mM Tris pH 7.5, 10 mM MgCl2, 0.05% NP-40).

All following steps were performed as described in [32]. In brief: immunoprecipitated crosslinked RNA-protein complexes were subjected to several enzymatic reactions on-bead. Subsequent de-phosphorylation of RNA 3′-ends by phosphatase-treatment, a 3′-RNA linker ligation and ^32^P-5′-end labelling of the RNA using T4 polynucleotide kinase and gamma-[^32^P]-ATP were performed. Complexes were resolved on a denaturing neutral-pH SDS-polyacrylamide gel electrophoresis (NuPAGE, Invitrogen), and transferred to a nitrocellulose membrane. Protein-RNA-complexes were visualized by autoradiography on an X-ray film at − 80 °C. Complexes of adequate size were excised from the membrane and RNA was isolated by proteinase K treatment. iCLIP library preparation was performed as described in [32], and sequencing on an Illumina MiSeq system, 75 bp single-read.

Following oligonucleotides were used:

- 3′-RNA linker (L31): P-UGAGAUCGGAAGAGCGGUUCAG-Puromycin.
- Reverse transcription primers (containing random and experimental barcode,):
- R1clip P-NNAACCNNNAGATCGGAAGAGCGTCGTGgatcCTGAACCGC.
- R6clip P-NNCCGGNNNAGATCGGAAGAGCGTCGTGgatcCTGAACCGC.
- R9clip P-NNGCCANNNAGATCGGAAGAGCGTCGTGgatcCTGAACCGC.
- R10clip P-NNGACCNNNAGATCGGAAGAGCGTCGTGgatcCTGAACCGC.
- R13clip P-NNTCCGNNNAGATCGGAAGAGCGTCGTGgatcCTGAACCGC.
- R14clip P-NNTGCCNNNAGATCGGAAGAGCGTCGTGgatcCTGAACCGC.

These reverse transcription primers add the following barcodes to the 5′-end of the CLIP-tag sequences (reverse complementary to primer sequence, termed “BC1–6” in bioinformatics analyses):

- Rrp4 Replicate 1: primer R1 - BC1: NNNGGTTNN.
- Rrp4 Replicate 2: primer R9 - BC4: NNNTGGCNN.
- Rrp41 Replicate 1: primer R10 - BC2: NNNGGTCNN.
- Rrp41 Replicate 2: primer R13 - BC5: NNNCGGANN.
- Trx Replicate 1: primer R14 - BC3: NNNGGCANN.
- Trx Replicate 2: primer R6 - BC6: NNNCCGGNN.

The control Trx2, which was directly adjacent to the exosome samples on the membrane (Fig. 1d), had some contaminations from exosome-samples, while the Trx1 control had a very low number of cDNA reads and the highest proportion of unmapped reads (Additional file 2).

Two independent bioreactor cultures were used for the iCLIP experiment. Samples from these cultures and from a third bioreactor culture were used for RNA-Seq. No rRNA depletion was performed for any of the samples. Total RNA was sequenced by Vertis Biotechnologie AG, Freising, Germany.

### cDNA reads mapping

Reads resulting from Illumina sequencing (75 nt) carried a nine nucleotide barcode at the 5′ end. Barcodes were composed of a three-nucleotide random barcode, a four-nucleotide experimental barcode and a final two-nucleotide random barcode. Several tools from the FASTX Toolkit (version 0.0.14; http://hannonlab.cshl.edu/fastx_toolkit/index.html) were used for read processing. Due to bad sequencing quality of the first nucleotide, it was removed using fastx_trimmer (−f 2). Reads were subjected to a two-step quality filtering using fastq_quality_filter (−q 5 -p95 and -q 20 -p 90). The random barcode was utilized to remove PCR duplicates via fastx_collapser. 3′ adapter sequences were removed by fastx_clipper (−a TGAGATCGGA) and manually trimming of remaining nucleotides using awk (4.1.3). Fastx_trimmer (−f 3) was used to remove the first random barcode (2 nt). Separation of reads according to the experimental barcode was done with fastx_barcode_splitter.pl (−-bol --exact). Remaining barcodes (6 nt) were subsequently remove using fastx_trimmer (−f 7). Resulting sequences were mapped to the *S. solfataricus* P2 genome (Sulfolobus_solfataricus_p2.ASM700v1.dna.toplevel.fa; obtained from Ensembl Bacteria) via bowtie2 (2.3.4) (−-sensitive) [50, 51], separating mappable (−-no-unal) and non-mappable (−-un) reads in distinct outputs. Separate BAM and bedgraph files for forward and reverse mapping reads were created using samtools [52] and bedtools [53].

### Detection of presumable circular RNAs

A major part of the non-aligning reads contained significant amounts of sequence fragments from *S. solfataricus*. Therefore the occurrence of circRNAs was investigated. For the detection of circRNAs interacting with the archaeal exosome, we applied a strategy similar to the one of Danan et al. [39] that was already proven successful in *Sulfolobus*, a prokaryotic organism lacking introns in protein-coding genes. The central idea of circRNA detection is the identification of a circularization junction. Sequencing of such a junction will result in a chimeric read, mapping to the target genome in a non-linear pattern. Briefly, detection of circRNAs harbouring a ligation site can therefore be performed by identification of two adjacent blast results in a single read aligning in a permuted way to the reference genome.

For circRNA detection only unaligned reads were used, as circularization junctions can only be contained in the non-aligning reads. The reads were subject to a blastn (2.2.31+) [54] search (−word_size 20 -outfmt ‘6 qseqid qlen length qstart qend sstart send sstrand qseq sseq evalue pident nident mismatch’ -max_hsps 1) with the *S. solfataricus* P2 sequence acting as a reference, whereby a first blast result with a minimum length of 20 nt was obtained. For further analysis blast results were split according to strandedness. Furthermore, only those hits were kept that fulfilled the requirement of a non-aligned sequence within the read 5′ or 3′ to the blast result. Those reads were subsequently subjected to a second blastn search (−word_size 12 -outfmt ‘6 qseqid qlen length qstart qend sstart send sstrand qseq sseq evalue pident nident mismatch’ -strand minus / plus, for the respective strandedness). The blast results obtained had to fulfil two requirements: They were not allowed to be more than 4000 nt apart from the position of the first blast hit and only single positional results were permitted. This allowed for the combination of the second fragments with their firstly identified counterparts.

Circular RNA detection was also attempted using CIRI2 (v2.0.6) [41]. CIRI2 relies on alignments generated by bwa mem (0.7.12-r1039) (options used: -T 19 -t 8). The resulting sam file was subsequently analyzed utilizing CIRI2 (options used: -I, −O, −F) and Perl version v5.22.0.

### Detection of putative 3′-attachments

As with the detection of circRNAs, reads harbouring a 3′ attachment synthesized by the exosome can only be found within the set of non-aligning reads. In theory reads carrying a 3′ attachment must consist of a 5′ section, which maps perfectly to the organism’s genome while there 3′ attachment does not, preventing alignment of the entire read. Therefore detection of 3′ attachments shared considerable similarities with circRNA detection. Reads with a 5′ end alignment to the *S. solfataricus* sequence were identified. Potential circRNAs were excluded.

The nucleotide composition of the 3′ attachments of those reads was statistically assessed for all bases as well as for each position within the attachments. Data were summarized by custom Python 3 scripts and plotted using R (3.4.2).

### Evaluation of iCLIP crosslink sites

To evaluate the binding preference of the protein complex, iCLIP crosslink sites had to be determined. To consider only those reads with a high mapping quality and unique mapping position, reads with a MAPQ-score below 20 were removed (samtools view -q 20) [52]. Replicated experiments were subsequently merged into a single bam file (samtools merge), which were sorted (samtools sort) and indexed (samtools index). PureCLIP (1.0.4) served in determining the crosslink positions (only standard options were used: -i, −bai, −g, −o and -nt 4 to profit from multithreading) [55]. To analyse the distribution of crosslinks within genes, every annotated gene was split into deciles and the number of crosslinks per decile was counted using bedtools. In this analysis the RefSeq [56] annotation for GCF_000007005.1_ASM700v1 (RefSeq assembly accession: GCF_000007005.1 which is identical to NCBI Reference Sequence: NC_002754.1) served as template, as it offered a higher quality in the annotation of open reading frames (ORFs) compared to the respective Ensembl annotation (Sulfolobus_solfataricus_p2.ASM700v1.38.chromosome. Chromosome.gff3). Counting results were plotted using R. An identical analysis was performed with artificially annotated operons. Operon information for *S. solfataricus* was obtained from the DOOR database (http://161.117.81.224/DOOR3/, date of accession 2020.07.31) [57]. All genes belonging to an operon were used to create an artificial operon annotation. Finally, the distribution of crosslinks upstream and downstream to the beginning of all 5′- and 3′-UTRs were assessed. Therefore, all crosslinks at a specific distance were summarized (using custom scripts) either in 10 nucleotide bins or for every nucleotide within a 100 nucleotide window to each side of the respective UTR. To compare crosslink abundance between different samples, we normalized to the relative crosslink count of the respective bin, meaning percentage of crosslinks within the given bin.

The coverage ratio between iCLIP and RNA-Seq mappings for all coding sequences (CDS) and intergenic regions (IGRs) was assessed as follows: firstly a bed file containing all IGRs was creating using bedtools complement (v2.27.1) [58] and the gff annotation of the RefSeq entry GCF_000007005.1. The coverage at every intergenic position was determined using samtools depth (1.7) (options: -a -b IGRs.bed) [52]. The resulting coverage data were further processed using R (3.4.4) and visualized by the bultin boxplot function.

Motif discovery for a predominant crosslink peak was performed using meme (−dna -nmotifs 1 -minw 4 -maxw 10) (4.12.0) [59]. Ten basepairs upstream and downstream of the respective crosslink site were extracted with samtools faidx (1.7) and supplied to meme as input data.

### Analysis of the nucleotide content of coding sequences

A-content, AG-content and the length of the longest poly(A) stretch were determined for each coding sequence (CDS) based on the RefSeq genome annotation of *Sulfolobus solfataricus* P2 (NCBI Reference Sequence: NC_002754.1) [56] using customized Perl scripts. Boxplots were plotted using R.

## Supplementary Information

**Additional file 1: Figure S1.** Uncropped images. **Figure S2.** CircRNAs identified in the exosome iCLIP analysis *of S. solfataricus.* **Figure S3.** Distribution of bases in RNA-tails detected in the aRrp41-iCLIP of the archaeal exosome. **Figure S4.** Poly(A) stretches in RNA are bound by the exosome. **Figure S5.** Global analysis of RNAs enriched by coimmunoprecipitation with the archaeal exosome by iCLIP. **Figure S6.** Antisense RNAs as preferred substrates of the archaeal exosome. **Figure S7.** Binding of the archaeal exosome to 5’and 3’parts of selected genes. **Figure S8.** Low affinity of the archaeal exosome to the abundant mRNA *tmoA* (SSO_RS06040 gene encoding toluene-4-monooxygenase system protein). **Figure S9.** Distribution of read counts in the genome of *S. solfataricus* was analyzed with respect to biotype RNA. **Figure S10.** Clustering of read counts at the 5′-and 3′-end of protein coding genes. **Figure S11.** Clustering of crosslink sites at the 5′ and 3′ end of predicted operons. **Figure S12.** Distribution of crosslink sites around the ends of annotated genes

**Additional file 2:.** iCLIP and RNA-Seq statistics.

**Additional file 3:.** Mapped crosslink sites.

**Additional file 4: **CircRNAs identified in the iCLIP analysis of the *S. solfataricus* exosome.

**Additional file 5:** RNA-tails with mapped 5′-sequence, detected by iCLIP of the archaeal exosome.

**Additional file 6: **Nucleotide content of the linear non-mapped cDNA reads in the iCLIP analysis of *S. solfataricus*.

**Additional file 7:** List of gene products, A-content, AG-content and the length of the longest poly(A) stretch for each coding sequence.

**Additional file 8:** Poly(A) stretches in CDS and in intergenic regions (IGRs), and binding of the archaeal exosome detected by iCLIP.

**Additional file 9:** iCLIP and RNA-Seq counts of annotated genes included in Fig. S5.

## Data Availability

The RNA-Seq datasets supporting the conclusions of this article are available in the NCBI’s Gene Expression Omnibus repository [60]; [accession number GSE149143] (https://www.ncbi.nlm.nih.gov/projects/geo/query/acc.cgi?acc=GSE149143). The code to perform and reproduce data analysis was deposited in the GitLab repository https://gitlab.com/sulfolobus/iclip.

## Abbreviations

A-content: Adenine-content
aCsl4: Archaeal Csl4 protein
aDnaG: Archaeal DnaG-like protein
aNop5: Archaeal Nop5 protein
A-rich: Adenine-rich
aRrp: Archaeal Rrp protein
asRNA: Antisense RNA
CDS: Coding sequence
circRNA: Circular RNA
CLIP: Crosslinking and immunoprecipitation
CoIP: Co-immunoprecipitation
dsRNA: Double-stranded RNA
iCLIP: Individual-nucleotide resolution UV crosslinking and immunoprecipitation
IGB: Integrated genome browser
IGR: Intergenic region
PNPase: Polynucleotide phosphorylase
Poly(A): Polyadenosine
RNA-Seq: RNA sequencing
rNDP: Ribonucleoside-diphosphate
RT-PCR: Reverse transcriptase-polymerase chain reaction
ssRNA: Single-stranded RNA
Trx: Thioredoxin
UTR: Untranslated region
