# Supplementary material for: iCLIP analysis of RNA substrates of the archaeal exosome
Source: BMC Genomics. 2020 Nov 16;21:797. doi: 10.1186/s12864-020-07200-x (PMC7667871; doi:10.1186/s12864-020-07200-x)
Supplement: Supplementary file 1 — Additional file 1: Figure S1. Uncropped images. Figure S2. CircRNAs identified in the exosome iCLIP analysis of S. solfataricus. Figure S3. Distribution of bases in RNA-tails detected in the aRrp41-iCLIP of the archaeal exosome. Figure S4. Poly(A) stretches in RNA are bound by the exosome. Figure S5. Global analysis of RNAs enriched by coimmunoprecipitation with the archaeal exosome by iCLIP. Figure S6. Antisense RNAs as preferred substrates of the archaeal exosome. Figure S7. Binding of the archaeal exosome to 5’and 3’parts of selected genes. Figure S8. Low affinity of the archaeal exosome to the abundant mRNA tmoA (SSO_RS06040 gene encoding toluene-4-monooxygenase system protein). Figure S9. Distribution of read counts in the genome of S. solfataricus was analyzed with respect to biotype RNA. Figure S10. Clustering of read counts at the 5′-and 3′-end of protein coding genes. Figure S11. Clustering of crosslink sites at the 5′ and 3′ end of predicted operons. Figure S12. Distribution of crosslink sites around the ends of annotated genes [file 12864_2020_7200_MOESM1_ESM.pdf]

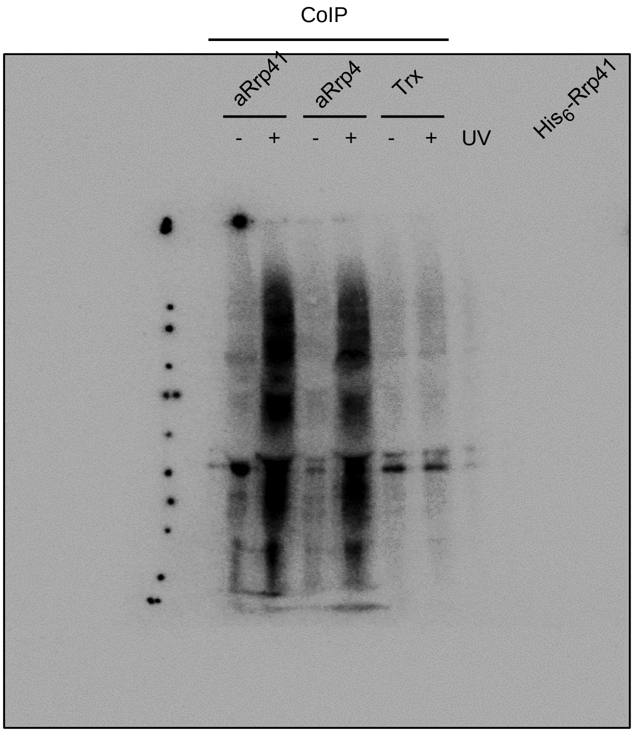

Related to Fig. 1A: Uncropped autoradiogram of a nitrocellulose membrane with transferred, coimmunoprecipitated protteins, attached to cross-linked, radioactively labelled RNA. On the left, positions of marker proteins was marked.

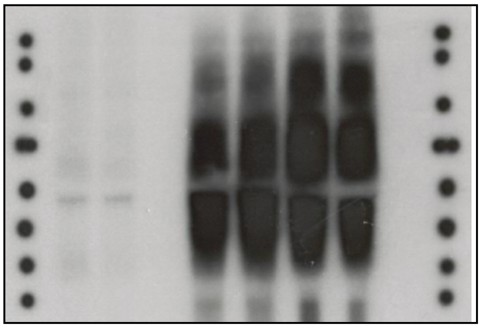

Related to Fig. 1D: Uncropped autoradiogram of the nitrocellulose membrane with samples used in our iCLIP analysis. On the left and on the rigth, positions of marker proteins were marked.

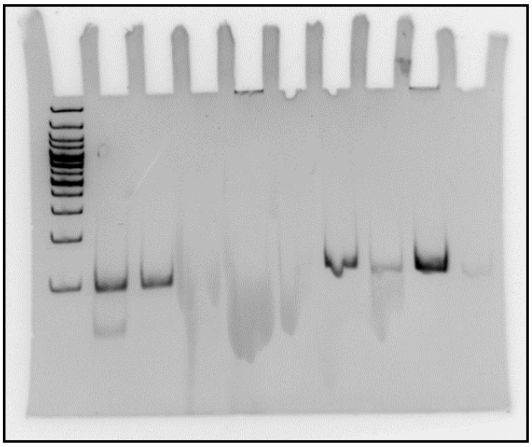

Related to Fig. 3C:
Picture showing the original gel of Fig. 2C with RT-PCR results and controls.

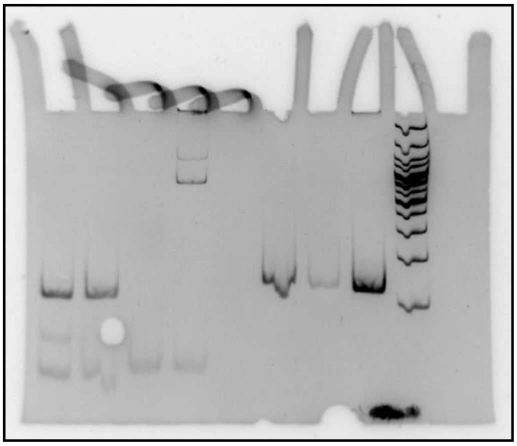

Related to Fig. 3D:
Picture showing the original gel of Fig. 2D with RT-PCR results and controls.

Figure S1. Uncropped images

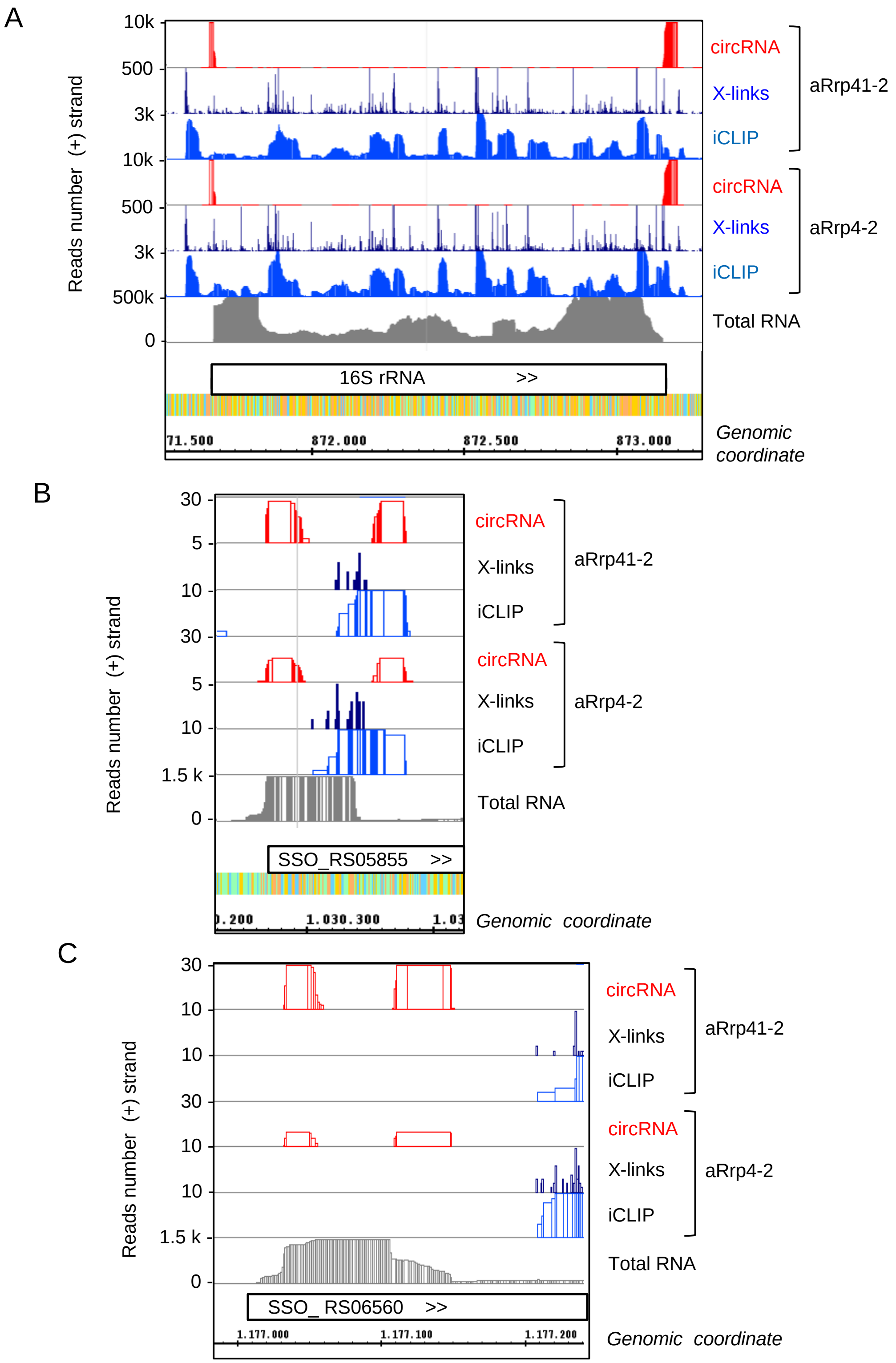

Figure S2. CircRNAs identified in the exosome iCLIP analysis *of S. solfataricus.* cDNA reads at the 16S rRNA locus **(A)** and at the 5'-regions of the two indicated transposase genes **(B) and (C)** are shown as coverage plots. Total RNA: cDNA reads of RNA-Seq of total RNA; iCLIP: mapped cDNA reads of the iCLIP analysis; crosslinks: corresponding mapped crosslink sites; CircRNA: cDNA reads of circRNAs detected in this study. Shown are data for aRrp4-2 and aRrp41-2 (indicated). Annotated genes (white bars) are indicated. >>: transcript corresponding to the plus strand; <<: transcript corresponding to the minus strand. Panel C shows that in this iCLIP replicate, the circRNA of SSO_RS06560 was coimmunoprecipitated using both antibodies, although no reads were mapped in this region.

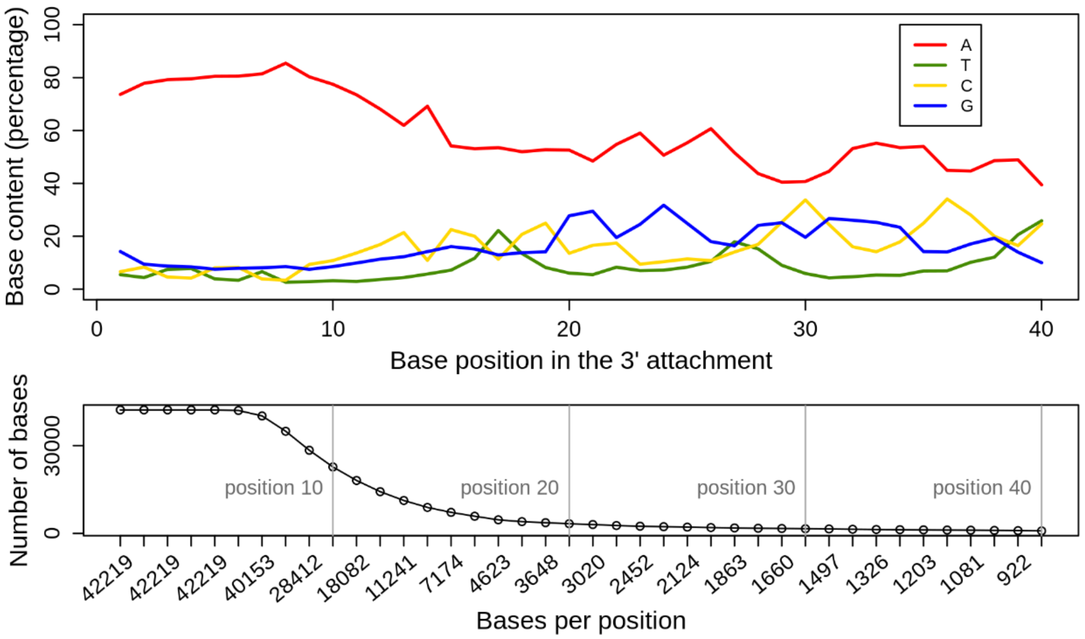

Figure S3. Distribution of bases in RNA-tails detected in the Rrp41-iCLIP of the archaeal exosome. The RNA-tails are posttranscriptional modifications at the 3'-end (3'-attachments presumably synthesized by the exosome without a template; [21]). The number of the analyzed bases per position is indicated. The maximal length of the analyzed tails was set to 40 nt.

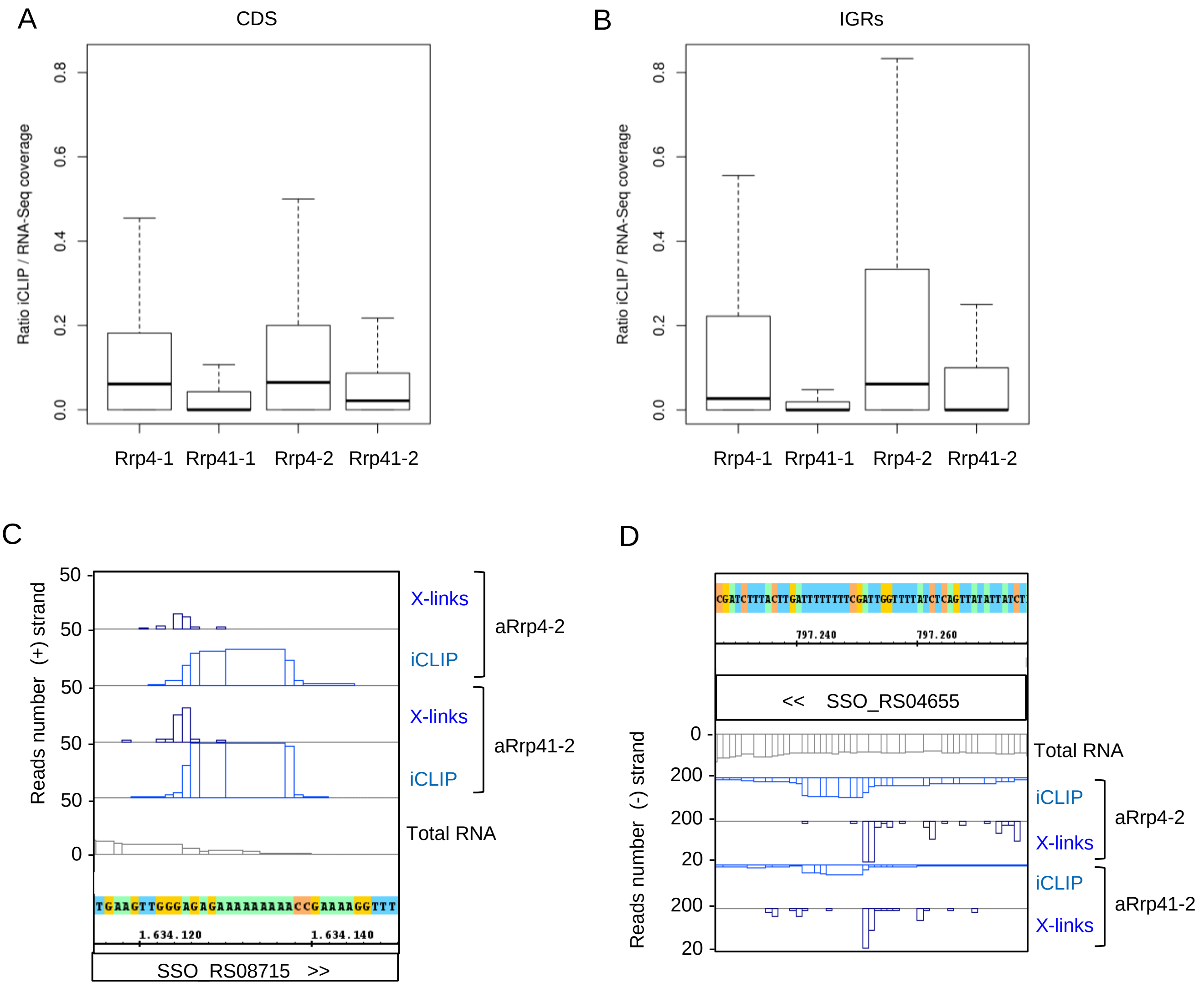

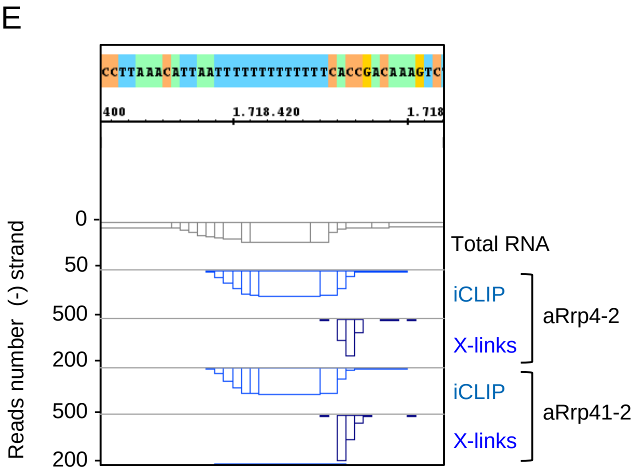

Figure S4. Poly(A) stretches in RNA are bound by the exosome. A) and **B)** Genome-wide analyses of the ratio of iCLIP to RNA-seq coverage in CDS and IGRs, respectively. Ratios for each CDS or IGR position in each of the iCLIP samples (indicated on the bottom) were determined. The result shows that generally, the RNA-Seq coverage was much higher than the iCLIP coverage. **C)** cDNA reads and crosslink sites in the SSO_RS08715 CDS encoding a transposase. **D)** cDNA reads and crosslink sites in the SSO_RS04655 CDS encoding glutamyl-tRNA(Gln) amidotransferase subunit D. **E)** cDNA reads and crosslink sites in the intergenic region between the convergently transcribed genes SSO_RS09125 (on the plus strand, encodes a hypothetical protein) and SSO_RS09130 (on the minus strand, encodes energy-coupling factor transporter transmembrane protein EcfT). Shown are data for aRrp4-2 and aRrp41-2 (indicated). For other descriptions see Figure S2.

Figure S5

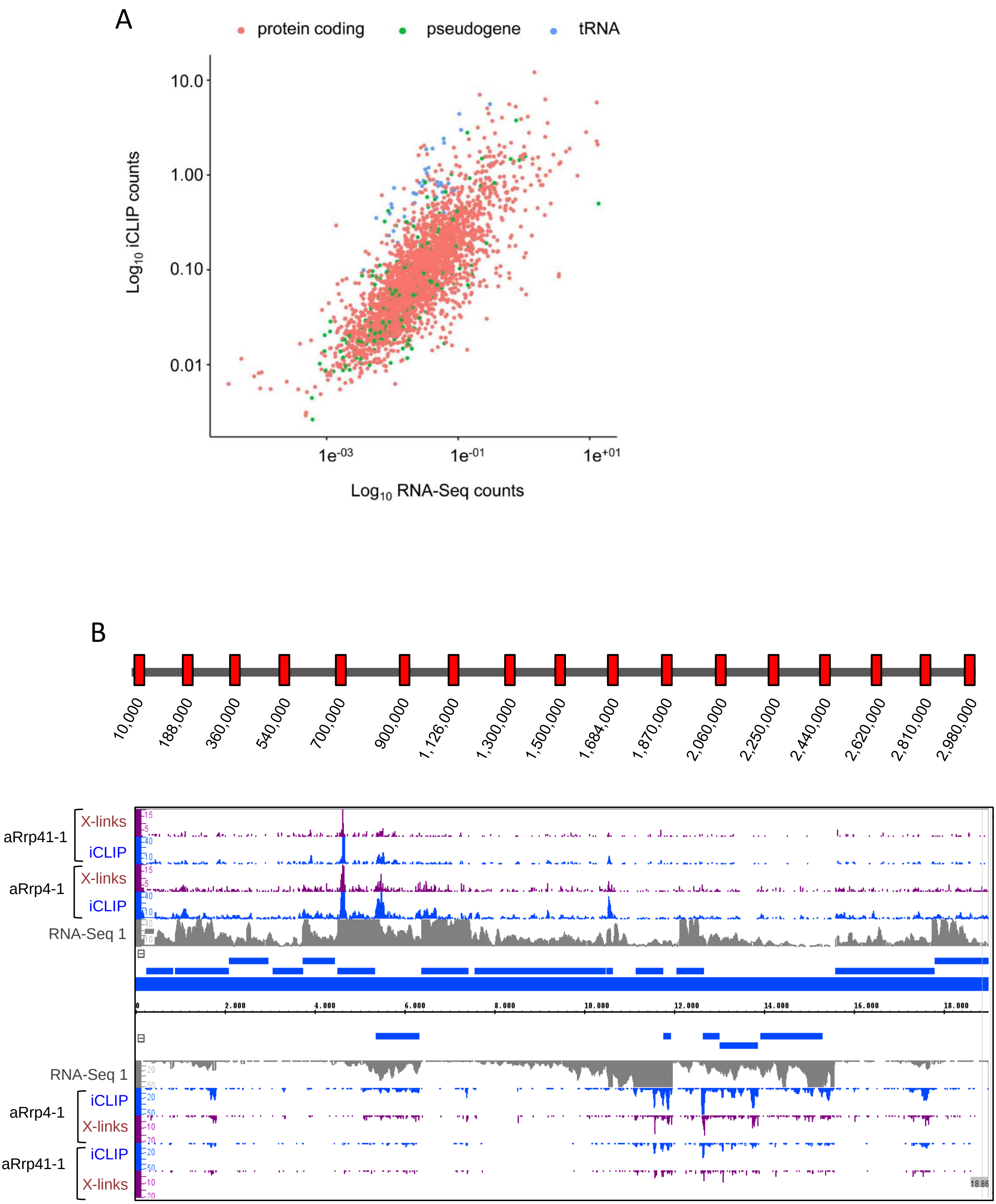
A
protein coding
pseudogene
tRNA
10.0
1.00
0.10
0.01
Log10 iCLIP counts
1e-03
1e-01
1e+01
Log10 RNA-Seq counts
B
10,000
188,000
360,000
540,000
700,000
900,000
1,126,000
1,300,000
1,500,000
1,684,000
1,870,000
2,060,000
2,250,000
2,440,000
2,620,000
2,810,000
2,980,000
aRrp41-1
X-links
iCLIP
aRrp4-1
X-links
iCLIP
RNA-Seq 1
2.000
4.000
6.000
8.000
10.000
12.000
14.000
16.000
18.000
RNA-Seq 1
aRrp4-1
iCLIP
X-links
aRrp41-1
iCLIP
X-links
18.86

Figure S5

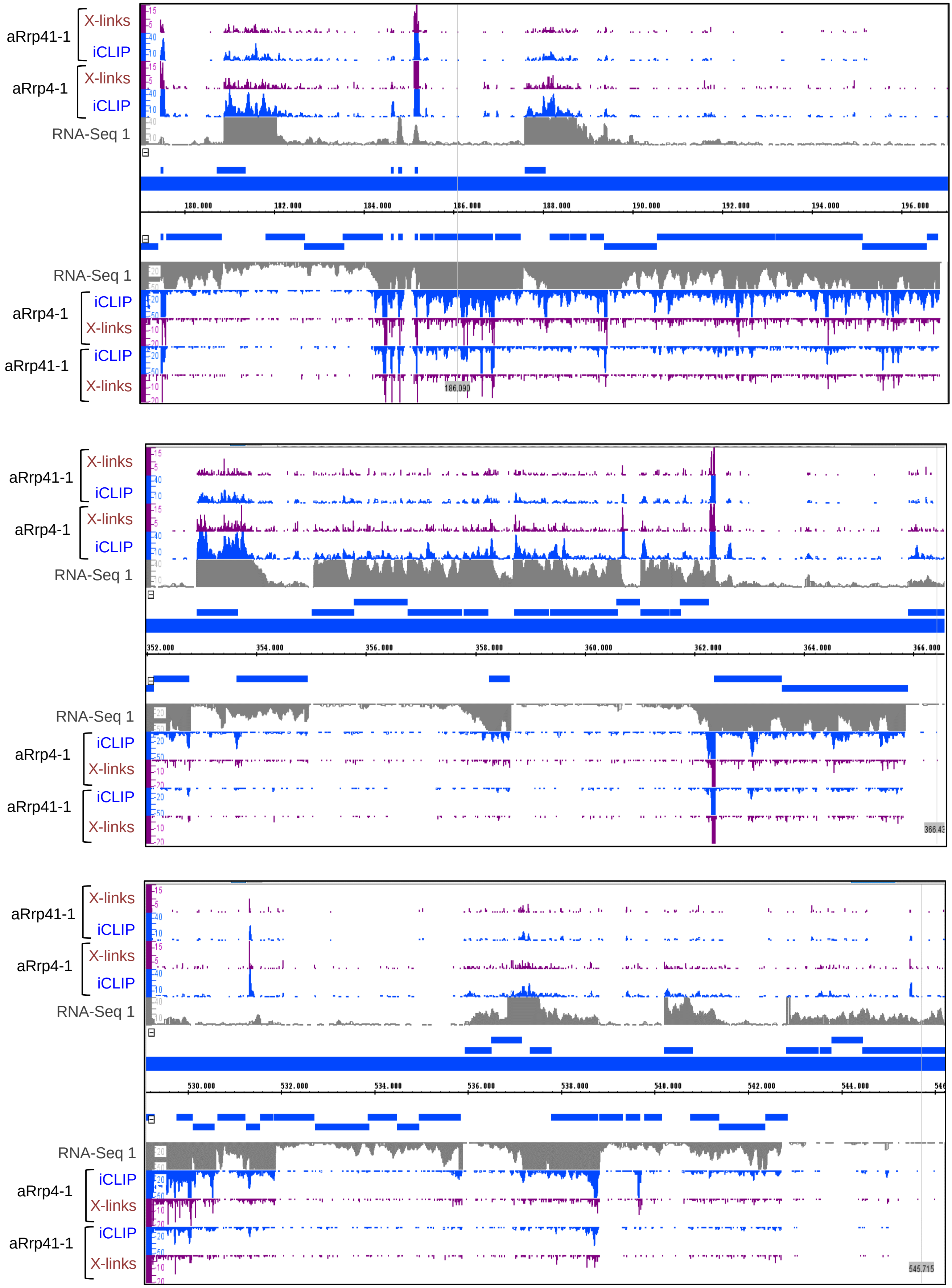

aRrp41-1
X-links
iCLIP
aRrp4-1
X-links
iCLIP
RNA-Seq 1
180.000
182.000
184.000
186.000
188.000
190.000
192.000
194.000
196.000
RNA-Seq 1
aRrp4-1
iCLIP
X-links
aRrp41-1
iCLIP
X-links
186.090
352.000
354.000
356.000
358.000
360.000
362.000
364.000
366.000
366.43
530.000
532.000
534.000
536.000
538.000
540.000
542.000
544.000
545.715

Figure S5

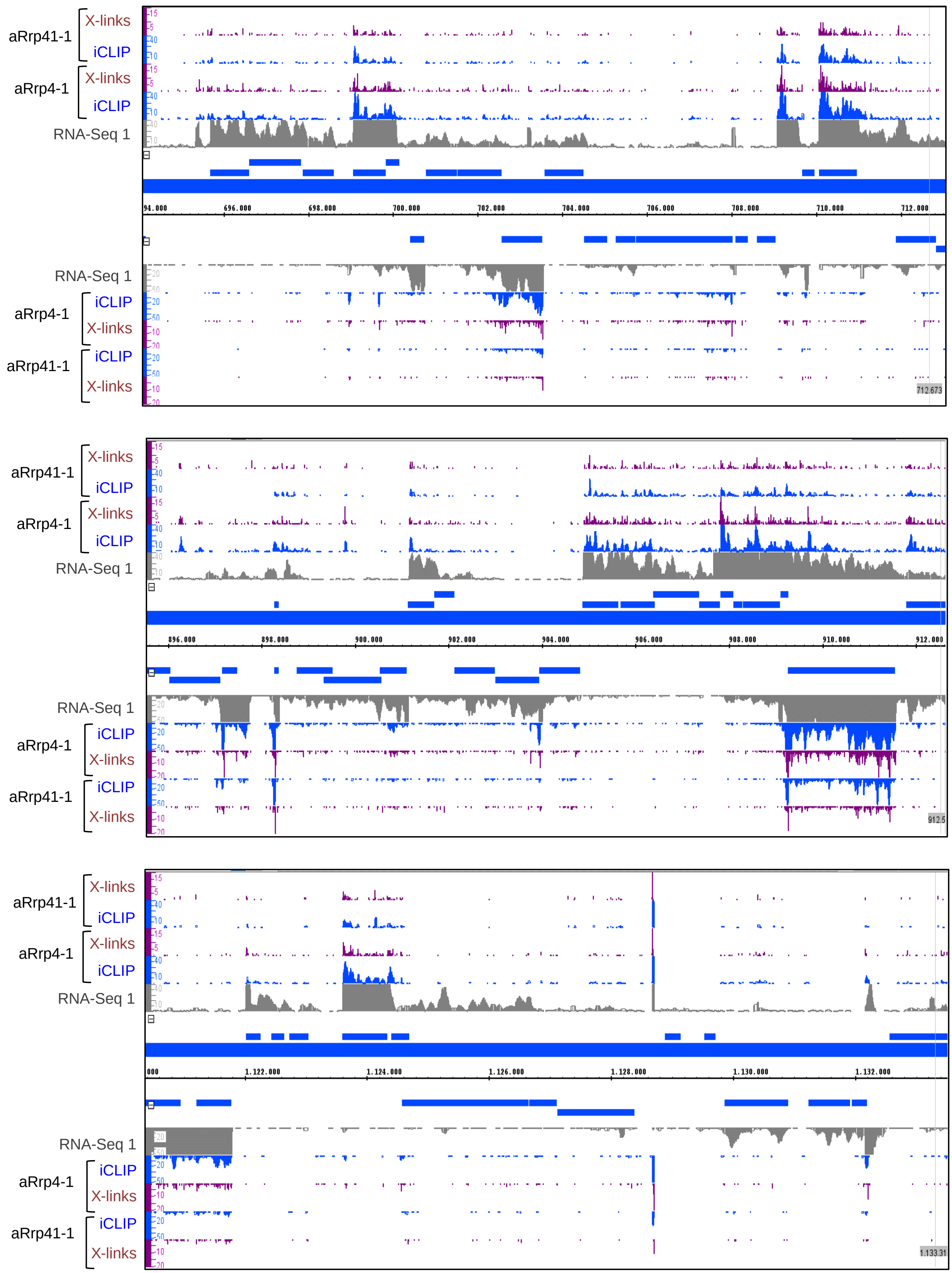

aRrp41-1
X-links
iCLIP
aRrp4-1
X-links
iCLIP
RNA-Seq 1
RNA-Seq 1
aRrp4-1
iCLIP
X-links
aRrp41-1
iCLIP
X-links

Figure S5

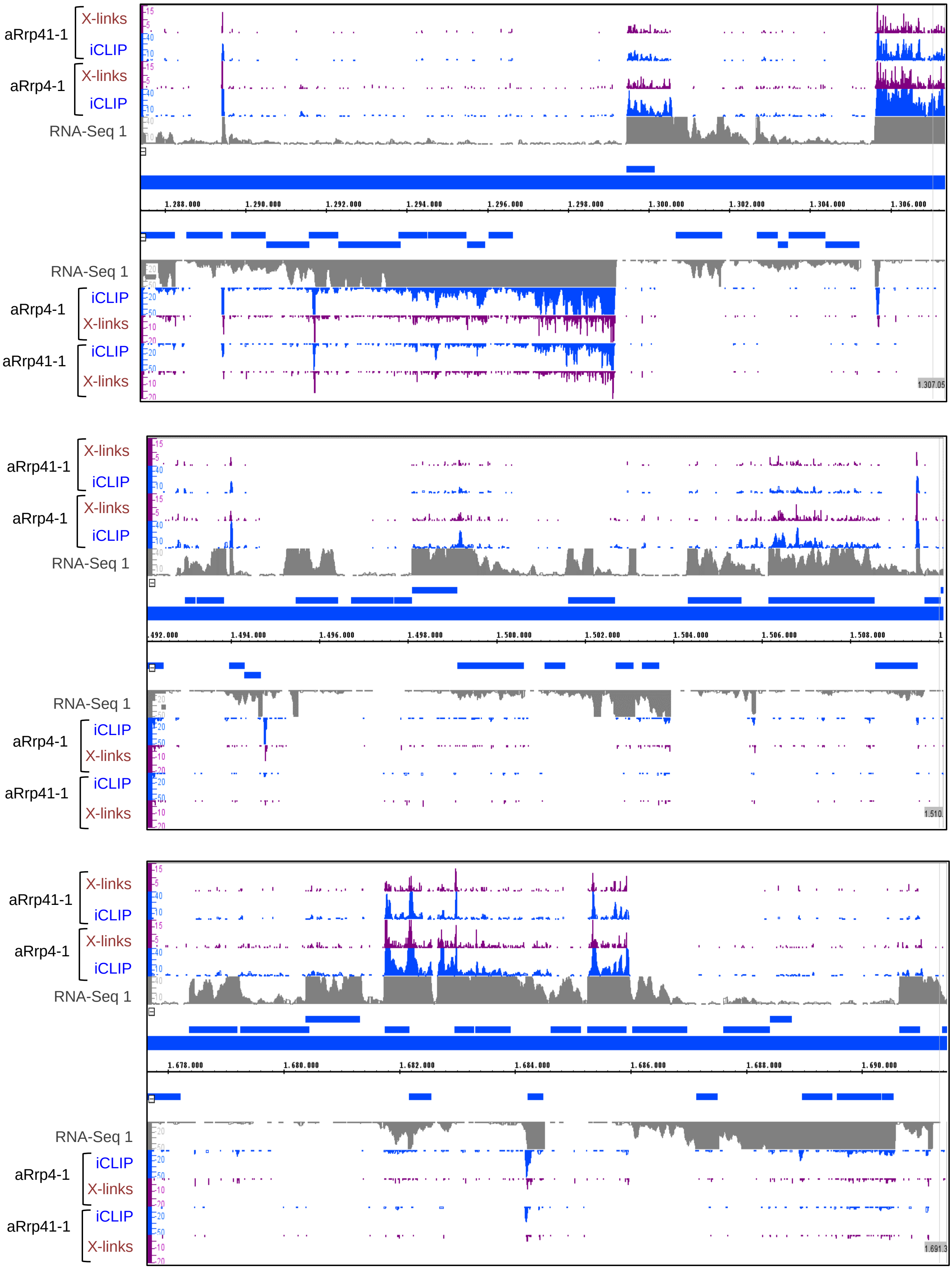
aRrp41-1
X-links
iCLIP
aRrp4-1
X-links
iCLIP
RNA-Seq 1
1.288.000
1.290.000
1.292.000
1.294.000
1.296.000
1.298.000
1.300.000
1.302.000
1.304.000
1.306.000
RNA-Seq 1
aRrp4-1
iCLIP
X-links
aRrp41-1
iCLIP
X-links
1.307.05
aRrp41-1
X-links
iCLIP
aRrp4-1
X-links
iCLIP
RNA-Seq 1
1.494.000
1.496.000
1.498.000
1.500.000
1.502.000
1.504.000
1.506.000
1.508.000
RNA-Seq 1
aRrp4-1
iCLIP
X-links
aRrp41-1
iCLIP
X-links
aRrp41-1
X-links
iCLIP
aRrp4-1
X-links
iCLIP
RNA-Seq 1
1.678.000
1.680.000
1.682.000
1.684.000
1.686.000
1.688.000
1.690.000
RNA-Seq 1
aRrp4-1
iCLIP
X-links
aRrp41-1
iCLIP
X-links

Figure S5

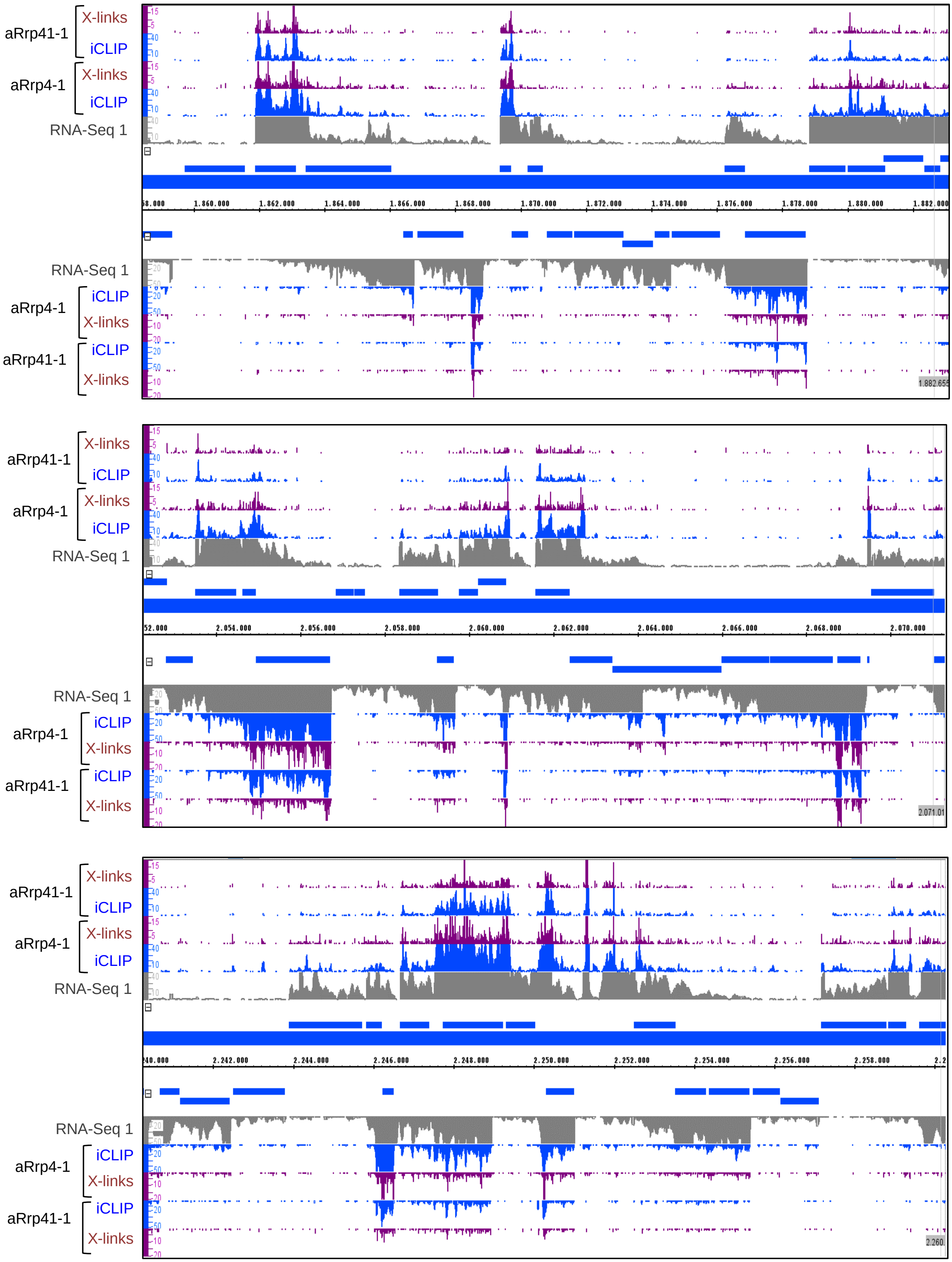

aRrp41-1
X-links
iCLIP
aRrp4-1
X-links
iCLIP
RNA-Seq 1
1.860.000
1.862.000
1.864.000
1.866.000
1.868.000
1.870.000
1.872.000
1.874.000
1.876.000
1.878.000
1.880.000
1.882.000
RNA-Seq 1
aRrp4-1
iCLIP
X-links
aRrp41-1
iCLIP
X-links
1.882.655
aRrp41-1
X-links
iCLIP
aRrp4-1
X-links
iCLIP
RNA-Seq 1
2.054.000
2.056.000
2.058.000
2.060.000
2.062.000
2.064.000
2.066.000
2.068.000
2.070.000
RNA-Seq 1
aRrp4-1
iCLIP
X-links
aRrp41-1
iCLIP
X-links
2.071.01
aRrp41-1
X-links
iCLIP
aRrp4-1
X-links
iCLIP
RNA-Seq 1
2.242.000
2.244.000
2.246.000
2.248.000
2.250.000
2.252.000
2.254.000
2.256.000
2.258.000
RNA-Seq 1
aRrp4-1
iCLIP
X-links
aRrp41-1
iCLIP
X-links
2.260.

Figure S5

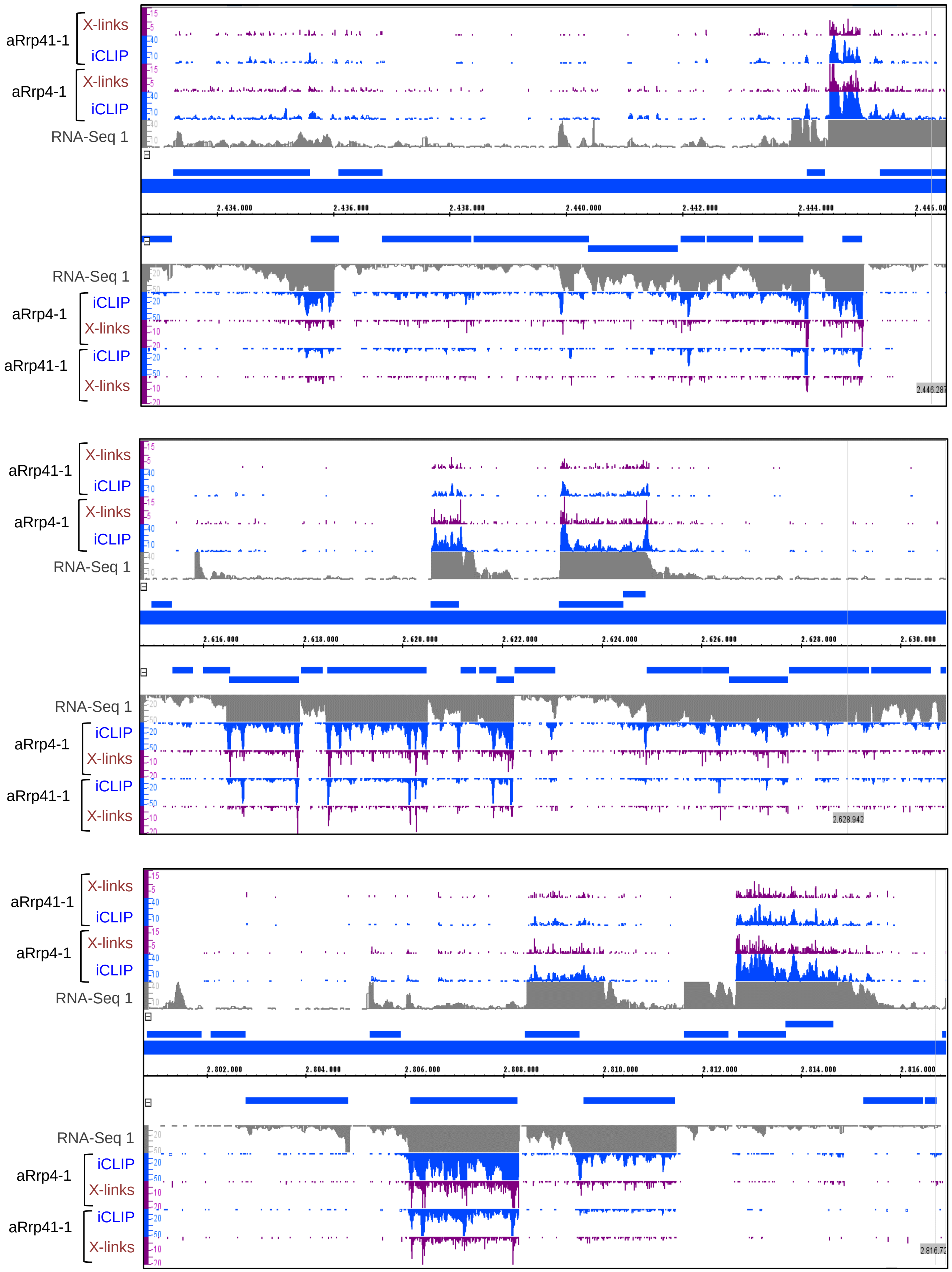
aRrp41-1
X-links
iCLIP
aRrp4-1
X-links
iCLIP
RNA-Seq 1
2.434.000
2.436.000
2.438.000
2.440.000
2.442.000
2.444.000
RNA-Seq 1
aRrp4-1
iCLIP
X-links
aRrp41-1
iCLIP
X-links
aRrp41-1
X-links
iCLIP
aRrp4-1
X-links
iCLIP
RNA-Seq 1
2.616.000
2.618.000
2.620.000
2.622.000
2.624.000
2.626.000
2.628.000
2.630.000
RNA-Seq 1
aRrp4-1
iCLIP
X-links
aRrp41-1
iCLIP
X-links
2.628.942
aRrp41-1
X-links
iCLIP
aRrp4-1
X-links
iCLIP
RNA-Seq 1
2.802.000
2.804.000
2.806.000
2.808.000
2.810.000
2.812.000
2.814.000
2.816.000
RNA-Seq 1
aRrp4-1
iCLIP
X-links
aRrp41-1
iCLIP
X-links

Figure S5

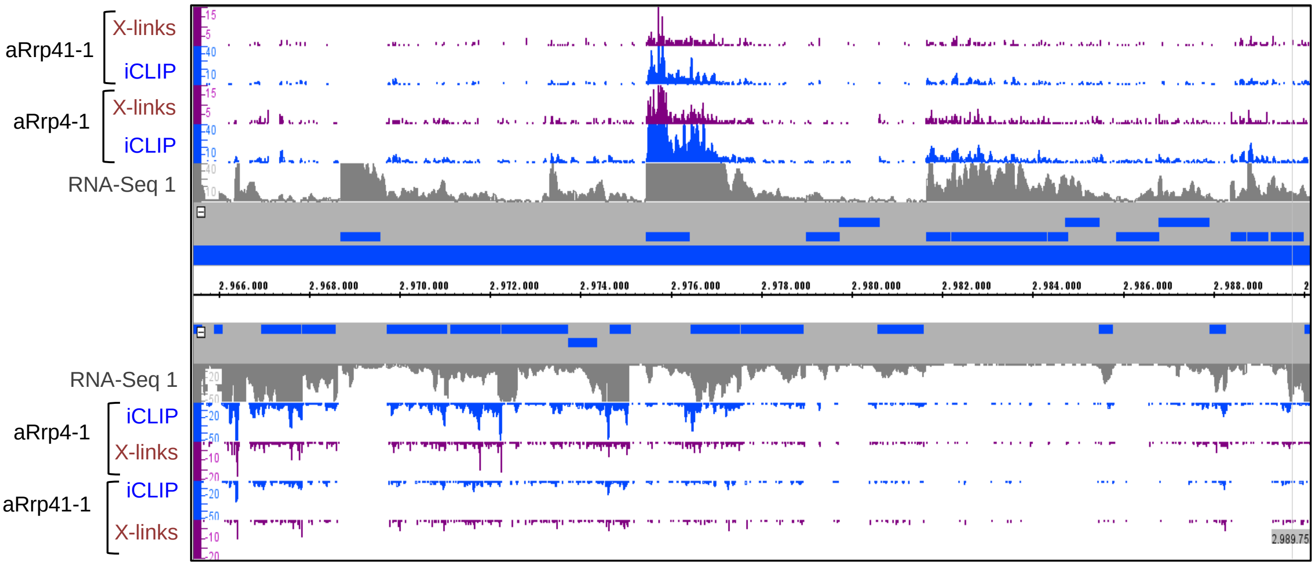

Figure S5. Global analysis of RNAs enriched by coimmunoprecipitation with the archaeal exosome by iCLIP. A) Transcriptome-wide comparison of iCLIP and RNA-Seq reads coverage. Analyzed gene types are indicated. The RNA-Seq read counts were TPM normalized. **B)** IGB-views of the iCLIP and RNA-Seq coverages, and of the mapped cross-link sites at random genomic regions. Above the IGB-views, the *S. solfataricus* chromosome (gray horizontal line) and the positions of the random regions (red boxes) are schematically shown. Chromosomal positions within the red box regions are indicated. The IGB views show the iCLIP coverage of Rrp4-1 and Rrp41-1 samples and of the RNA-Seq sample 1. For additional details, see Fig. S2.

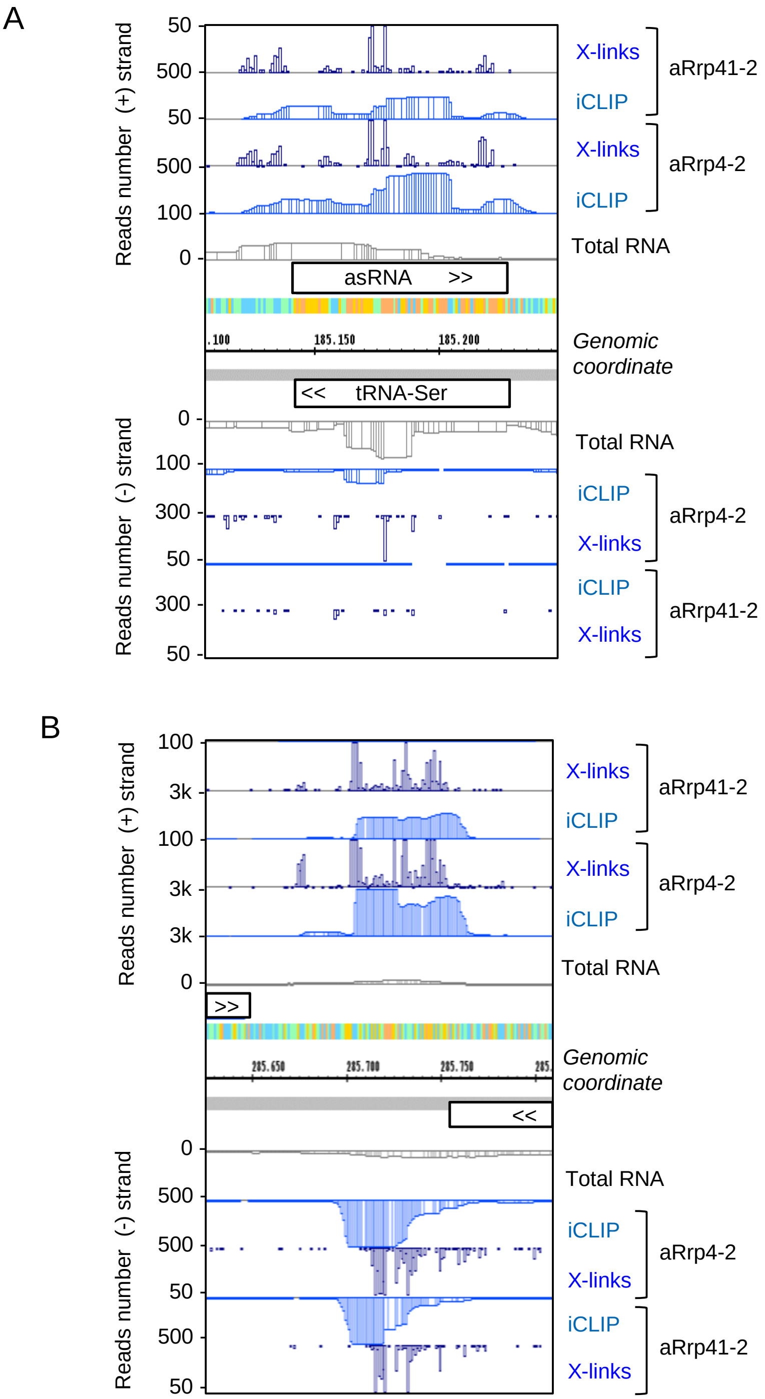

Figure S6. Antisense RNAs as preferred substrates of the archaeal exosome. A) cDNA reads and crosslink sites at the tRNA-Ser locus (SSO_RS01080, alias SSOt12) show strong enrichment of antisense RNA in the iCLIP. **B)** cDNA reads and crosslink sites in the intergenic region between two convergently transcribed genes (SSO_RS01650 encoding a hypothetical protein on the plus strand and SSO_RS01655 encoding tRNA 4-thiouridine(8) synthase ThiI on the minus strand) show strong iCLIP enrichment of potentially antisense transcripts corresponding to overlapping 3'-UTRs. Shown are data for aRrp4-2 and aRrp41-2 (indicated). For other descriptions see Figure S2.

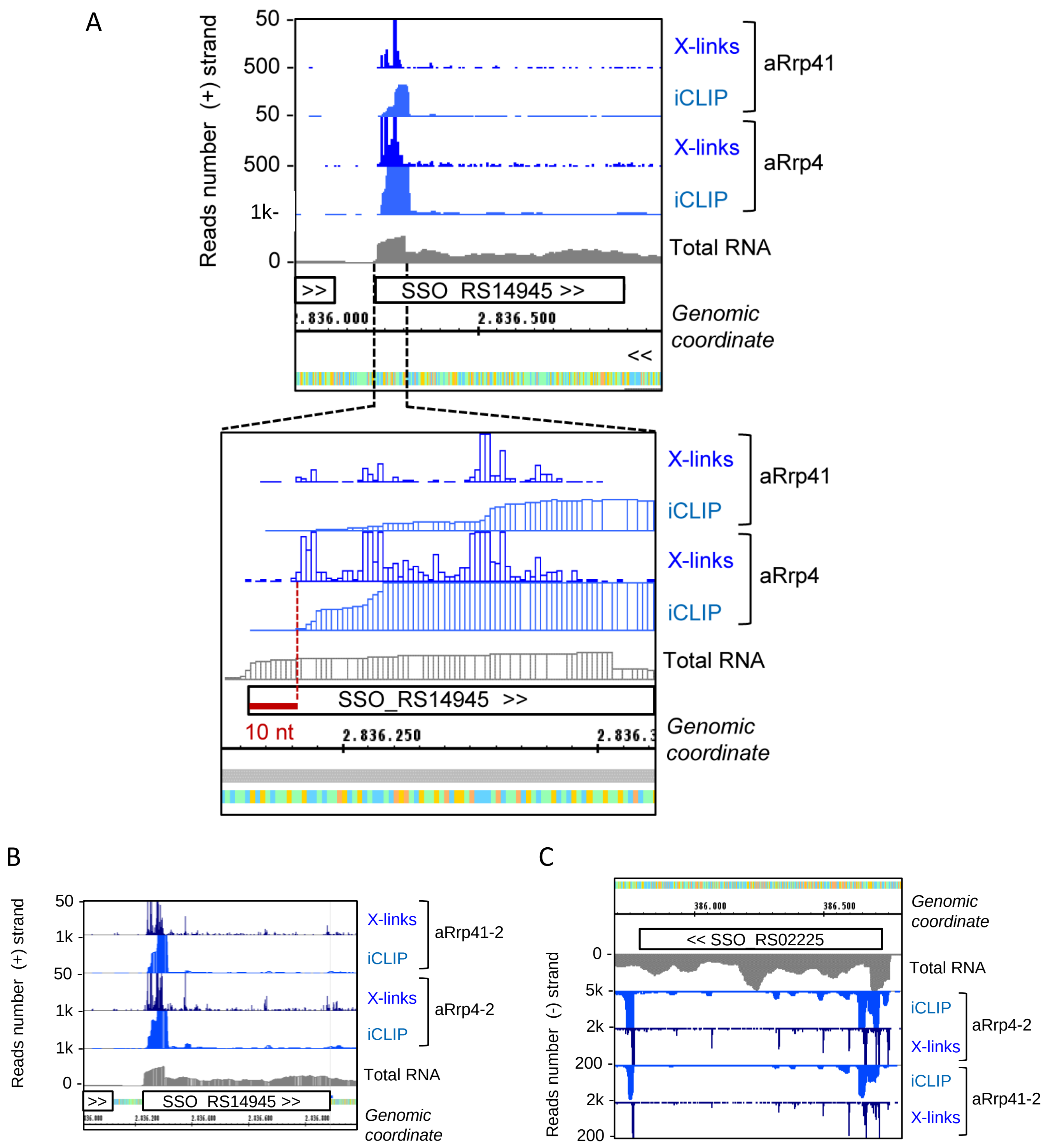

Figure S7. Binding of the archaeal exosome to 5‘ and 3‘ parts of selected genes. A) Binding to the 5’-part of SSO_RS14945. The top panel shows cDNA reads and crosslink sites of SSO_RS14945 (encodes a XRE family transcriptional regulator) and its flanking regions. A zoom in the 5’-part of the gene is shown in the bottom panel. The first 10 nt of the leaderless mRNA were not crosslinked (marked with red lines). Shown are data for aRrp41-1, aRrp4-1 and Trx-1 (indicated). **B)** Data for aRrp4-2 and aRrp41-2 (indicated) showing the exosome binding to the 5’-part of SSO_RS14945. **C)** Exosome binding to the 5’- and 3’-part of the SSO_RS02225 transcript. Shown are data for aRrp4-2 and aRrp41-2 (indicated). For other descriptions see Fig. S2.

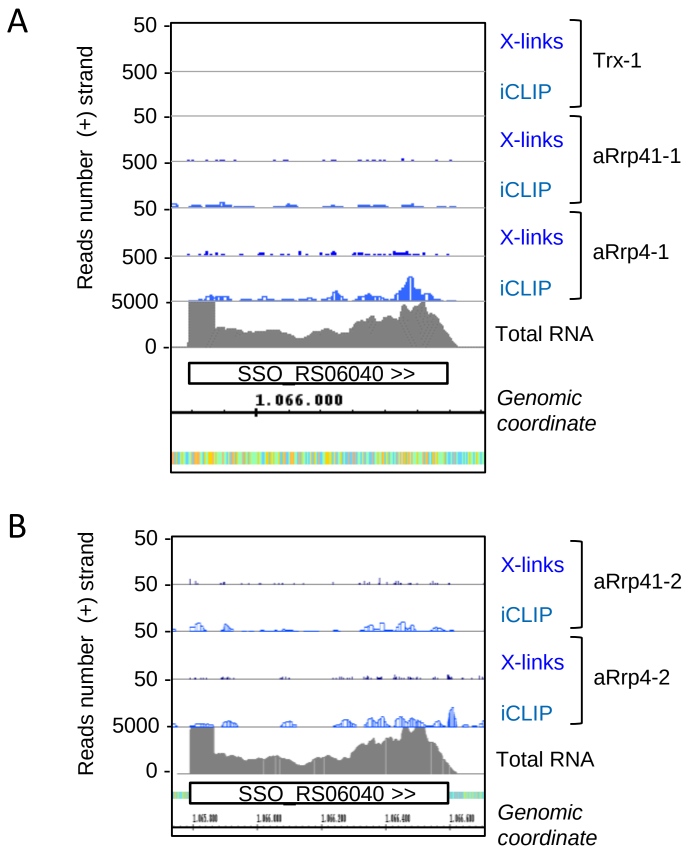

Figure S8. Low affinity of the archaeal exosome to the abundant mRNA *tmoA* (SSO_RS06040 gene encoding toluene-4-monooxygenase system protein A). Shown are results of the first (A) and second (B) iCLIP experiment. For other descriptions see Fig. S2.

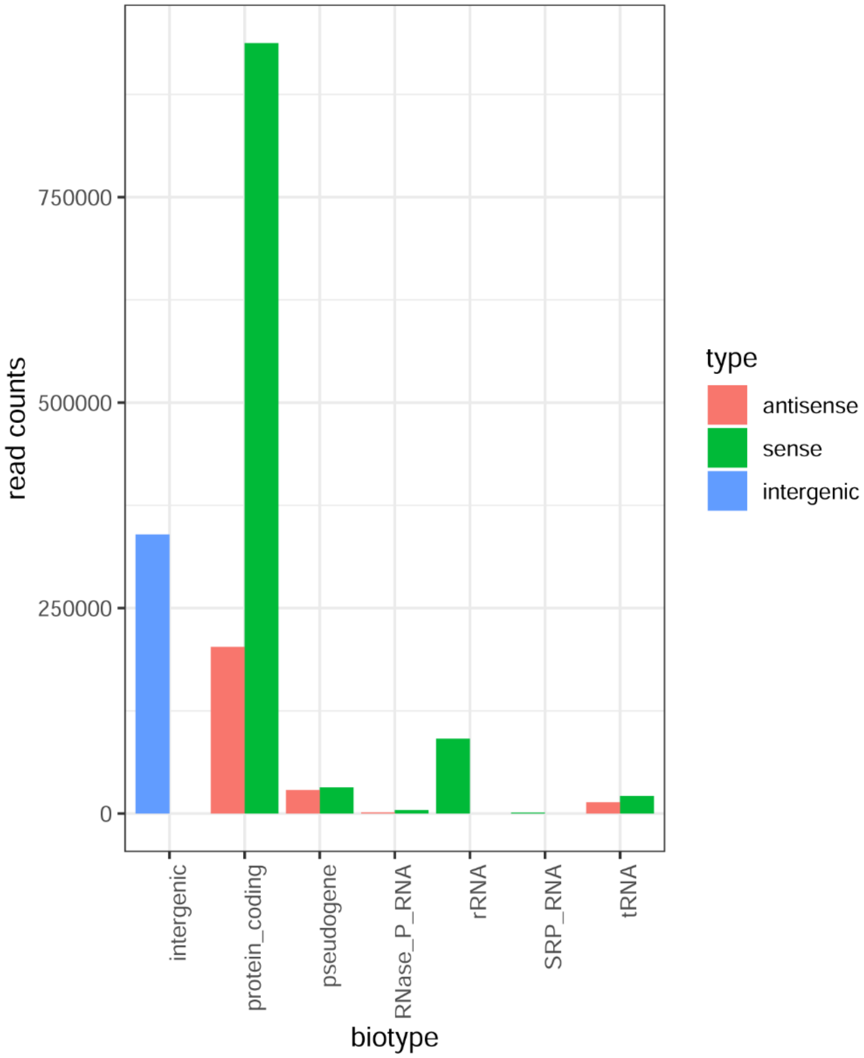

Figure S9. Distribution of read counts in the genome of *S. solfataricus* was analyzed with respect to biotype RNA.

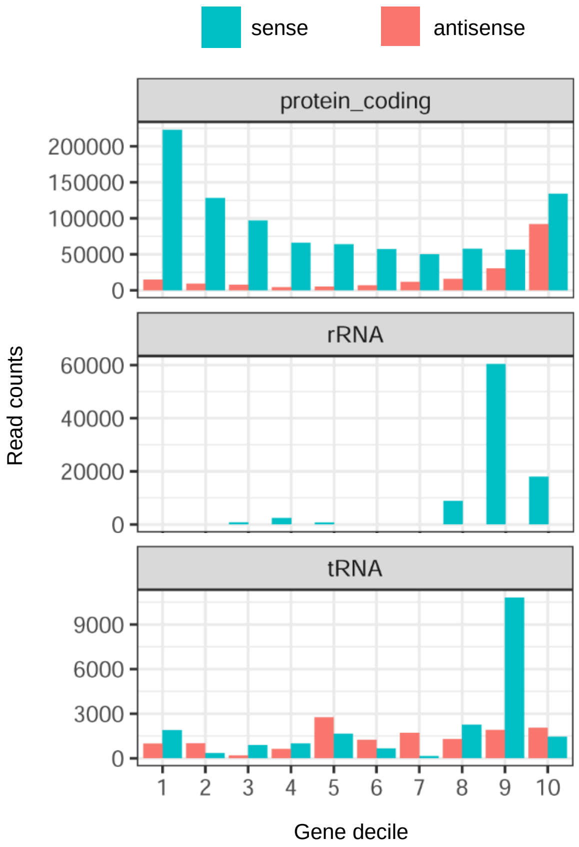

Figure S10. Clustering of read counts at the 5'- and 3'-end of protein coding genes. The read counts per decile of annotated genes was analyzed. Gene biotype and transcript orientation are indicated.

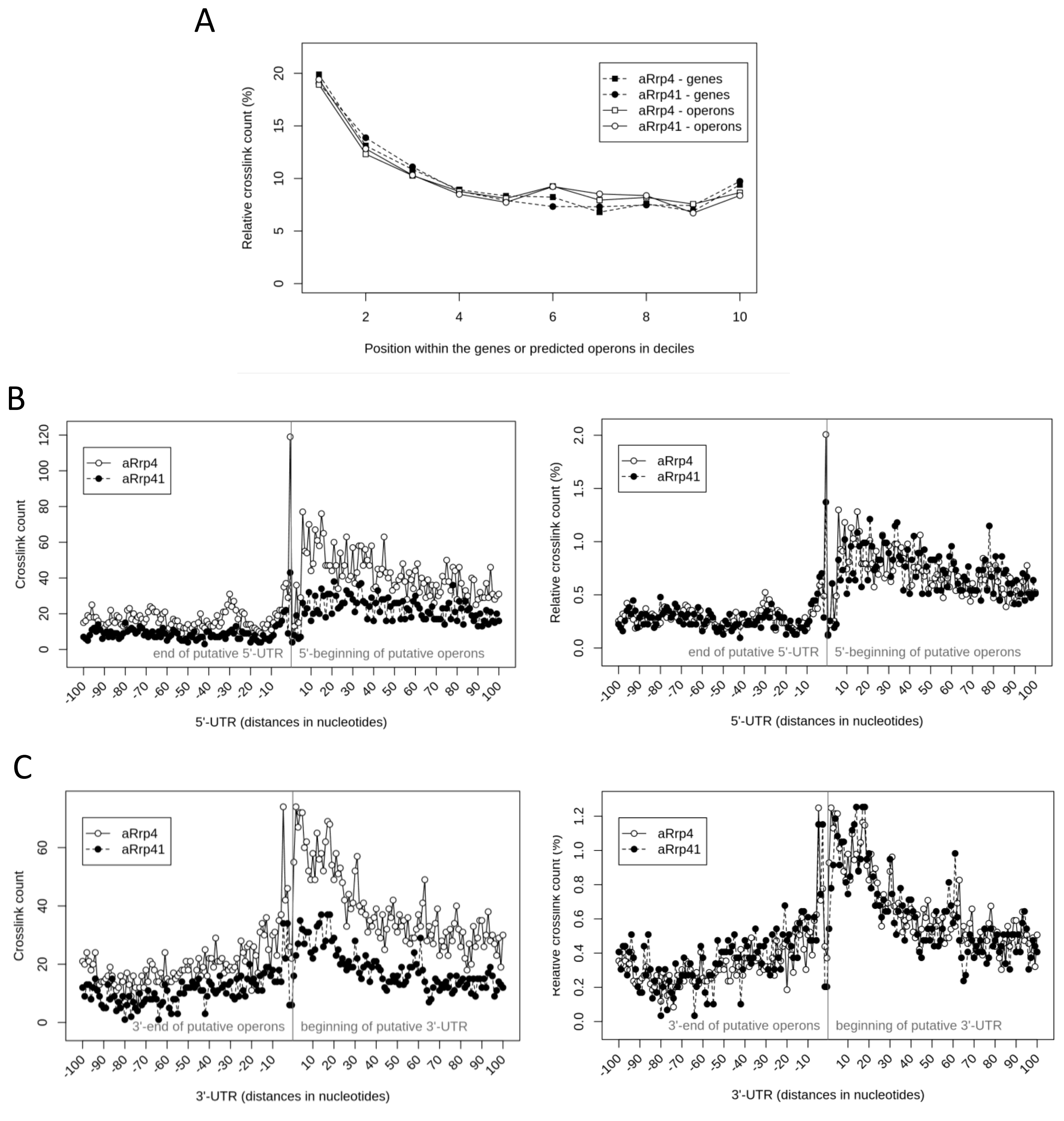

Figure S11. Clustering of crosslink sites at the 5' and 3' end of predicted operons. A) The crosslink sites per decile of predicted operons was analyzed. Negative nt positions correspond to 5'-UTRs, positive positions are located in the genes. 100 nt upstream and downstream of the gene starts were analyzed. **B)** Two alternative representations of the clustering at the 5'-end. **C)** Two alternative representations of the clustering at the 3'- end. Negative nt positions are located in the genes, positive positions correspond to the 3'-UTRs. 100 nt upstream and downstream of the gene ends were analyzed.

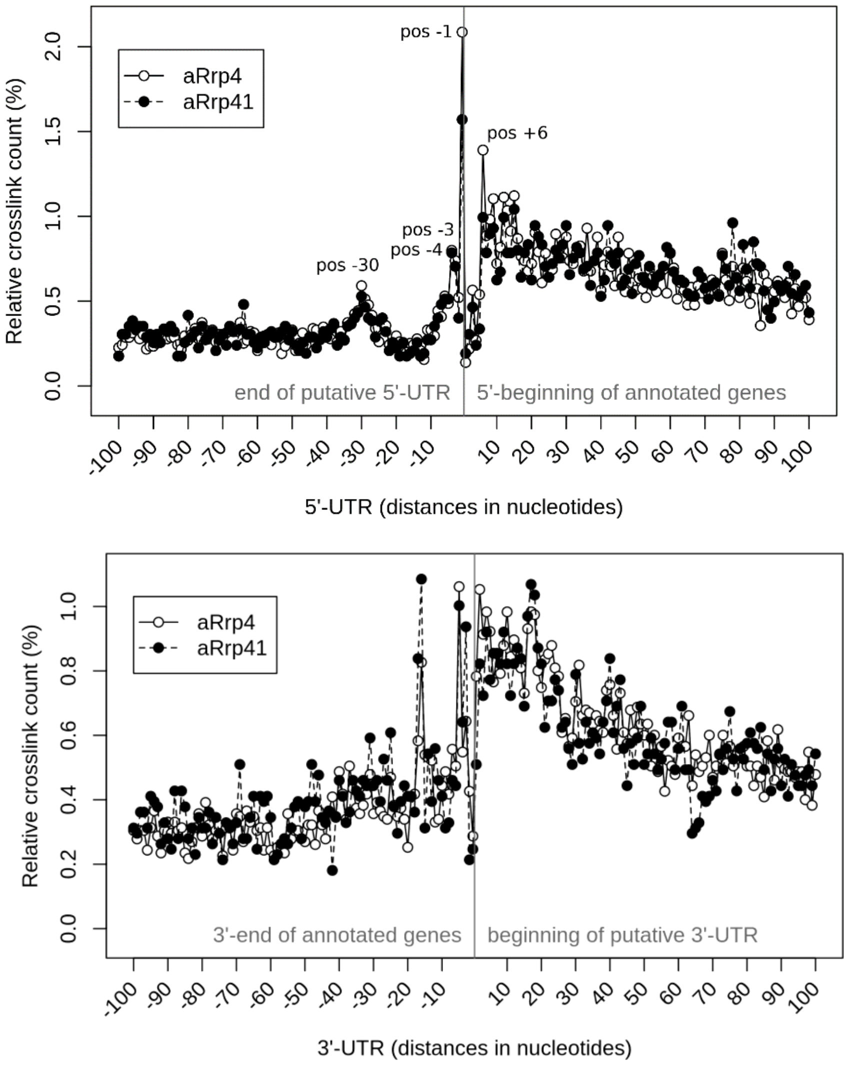

Figure S12. Distribution of crosslink sites around the ends of annotated genes. This figure shows an alternative representation of Fig. 10A and Fig. 10C. **A)** Positions of crosslink sites around the start of annotated genes. Negative nt positions correspond to 5'-UTRs, positive positions are located in the genes. 100 nt upstream and downstream of the gene starts were analyzed. **B)** Positions of crosslink sites around the end of annotated genes. Negative nt positions are located in the genes, positive positions correspond to the 3'-UTRs. 100 nt upstream and downstream of the gene ends were analyzed.
