# Supplementary material for: iCLIP analysis of RNA substrates of the archaeal exosome
Source: BMC Genomics. 2020 Nov 16;21:797. doi: 10.1186/s12864-020-07200-x (PMC7667871; doi:10.1186/s12864-020-07200-x)
Supplement: Supplementary file 6 — Additional file 6: Nucleotide content of the linear non-mapped cDNA reads in the iCLIP analysis of S. solfataricus. [file 12864_2020_7200_MOESM6_ESM.pdf]

Additional file 6. Nucleotide content of the linear non-mapped cDNA reads in the iCLIP analysis of *S. solfataricus*.

CoIP with	nt number	% A	% G	% C	% T	GC content	AG content
**Non-mapped reads**							
Rrp4(1)	793,417	73.4	12.1	8.9	5.6	21	85.5
Rrp4(2)	447,581	73	12.4	8.4	6.2	20.8	85.4
Rrp41(1)	170,542	73.7	11.9	8.5	5.9	20.4	85.6
Rrp41(2)	326,377	75	10	9.5	5.5	19.5	75
Trx(1)	3,480	44	20.1	18.7	17.2	38.8	64.1
